# Factors associated with clinical outcomes of breast cancer based on glucose metabolic activity of subcutaneous adipose tissue

**DOI:** 10.3389/fonc.2026.1722085

**Published:** 2026-02-23

**Authors:** Jinci Mai, Huanhua Wu, Wanwan Wu, Jinjun Zhou, Yuee Wu, Xiaobei Duan, Rizhao Wu, Youzhu Hu, Zuowu Zhen, Binhao Huang

**Affiliations:** 1 Department of Nuclear Medicine, Jiangmen Central Hospital, Jiangmen, Guangdong, China; 2 The Affiliated Shunde Hospital of Jinan University, Foshan, Guangdong, China; 3 Department of General Surgery, The Affiliated Shunde Hospital of Jinan University, Foshan, Guangdong, China

**Keywords:** breast cancer, positron emission tomography (PET), prognosis, subcutaneous adipose tissue, visceral adipose tissue

## Abstract

**Background:**

Obesity increases the risk of breast cancer, with dysfunctional metabolic activity in subcutaneous adipose tissue (SAT) implicated as a key underlying mechanism. This study sought to explore the correlation between SAT metabolic activity and clinical prognosis among patients with breast cancer.

**Methods:**

Body composition parameters at the level of the third lumbar vertebra and clinicopathological data from 74 patients with breast cancer were collected. Two Cox models were established using the least absolute shrinkage and selection operator (LASSO) for variable selection. The two models were compared using the time-dependent area under the receiver operating characteristic curve (AUC) and the net reclassification index (NRI). Furthermore, the confounding effects on the association among the mean standard uptake value of subcutaneous adipose tissue (SUVmean_SAT), American Joint Committee on Cancer (AJCC) stage, and progression status in patients with breast cancer were assessed.

**Results:**

Twenty-five out of 74 patients experienced recurrence during a median follow-up of 18 months. AJCC stage (*p* < 0.001) and SUVmean_SAT (*p* = 0.001) were significant independent prognostic factors. The AUC value and NRI of the combined Cox model (SUVmean_SAT plus AJCC stage) were higher than those of the Cox model based on AJCC stage alone. In addition, changes in the effect estimates showed a 12.2% decrease in hazard ratios when SUVmean_SAT was added to the AJCC stage model. High SUVmean_SAT was significantly associated with an increased risk of progression-free survival in patients with breast cancer.

**Conclusions:**

A comprehensive assessment incorporating both SUVmean_SAT and AJCC stage may enhance understanding of the impact of adiposity on breast cancer prognosis.

## Introduction

In many nations, obesity is becoming a serious public health issue (1). An increasing number of studies have shown that obesity increases the risk of breast, colorectal, liver, and other cancers (2, 3). Most research indicates that adipose inflammation and associated microenvironmental alterations are the mechanisms by which obesity drives tumor growth (4, 5).

^18^F-fluorodeoxyglucose positron emission tomography/computed tomography (^18^F-FDG PET/CT) is mainly used in the imaging of malignant tumors. It works via the glucose transporter 1 pathway, which allows cells to absorb FDG, an analog of glucose. After uptake by cells, FDG is phosphorylated by hexokinase, trapping FDG-6-phosphate within cells. As a result of this process, PET/CT can detect regions with elevated glucose metabolism and utilization, which is useful not only for malignant tumors but also for infection and inflammatory sites (6). Therefore, the standardized uptake value (SUV), a parameter of ^18^F-FDG PET/CT, can be utilized to evaluate the inflammatory environment of adipose tissue.

Few studies are available on this topic. According to a study conducted by Korean researchers, individuals with lymph node metastases of breast cancer had a significantly higher ratio of maximum standardized uptake value of visceral adipose tissue (SUVmax_VAT) to maximum standardized uptake value of subcutaneous adipose tissue (SUVmax_SAT) than those without metastases (7). In addition, it was reported that breast cancer patients with a high mean standardized uptake value of visceral adipose tissue (SUVmean_VAT) had a poor prognosis (8). In a study of breast cancer patients in Turkey, patients with a low ratio of SUV_VAT to SUV_SAT showed a poor response to neoadjuvant chemotherapy (9). Contrary to the findings of the Korean study, it has been suggested that breast adipose tissue is more closely associated with subcutaneous adipose tissue (SAT) than visceral adipose tissue (VAT) (10).

This was a retrospective cohort study of patients with breast cancer who underwent ^18^F-FDG PET/CT prior to treatment. The primary objective was to evaluate the prognostic significance of glucose uptake in SAT in breast cancer.

## Materials and methods

### Study population

**Figure 1:**
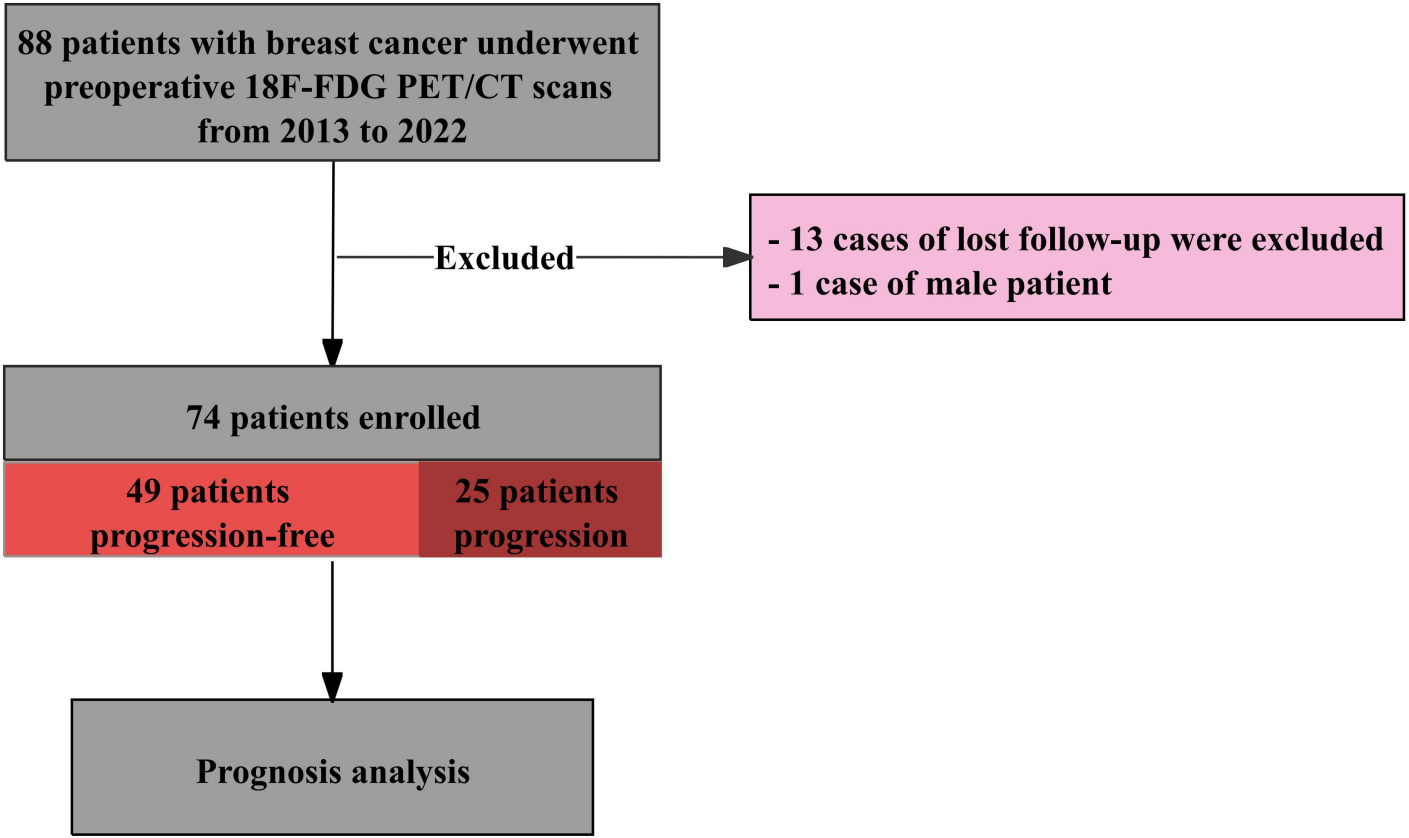
Schematic of the study design and outcomes.

This was a single-center retrospective study of 88 female patients with breast cancer undergoing pretreatment ^18^F-FDG PET/CT scans between January 2013 and December 2022. These patients did not have a previous history of other invasive cancers. After completing the scans, the patients received antitumor therapy. We excluded 14 patients due to loss to follow-up (*n* = 13) and one male patient (*n* = 1). The final analytic sample included 74 patients, of whom 25 (33.8%) experienced disease progression and 49 (66.2%) were progression-free, as shown in **Figure 1**. This study was approved by the Institutional Review Board (IRB), and an informed consent waiver was formally granted. All patient data were anonymized, with personal identifiers removed, and the study strictly adhered to the hospital’s privacy protection policies and the relevant provisions of the Declaration of Helsinki.

### ^18^F-FDG PET/CT protocol

The ^18^F-FDG PET/CT (GE Discovery VCT) scan covered the region from the skull vertex to the proximal thighs. The CT scan (120 kVp, automatic tube current tracking technology, and 3.75 mm slice thickness) was performed first for attenuation correction, followed by the PET scan. The PET scan (18 cm axial field of view with a 3.75-mm spatial resolution) was performed for nine bed positions at 1 min per bed position. The PET images were reconstructed by an iterative algorithm (three-dimensional row-action maximum likelihood algorithm) with an attenuation correction map derived from the CT scan. All patients had fasting serum glucose levels below 11.1 mmol/L.

### Follow-up and clinical outcome

Individual clinical and pathologic information in the study came from digital medical records. The outcome was progression-free survival (PFS). Follow-up time for PFS was calculated as the time from the start of treatment to cancer progression or the end of follow-up (31 December 2022), whichever occurred first. Tumor morphology was classified as mass/nodular type or diffuse type. Lymphovascular invasion was defined as the presence of tumor cells within a definite vessel and/or lymphatics surrounding the tumor in breast tissue. Molecular subtypes were luminal A, luminal B, HER-2-enriched, and triple-negative types. The Ki-67 antigen (Ki-67) index was classified as low (< 15%) or high (> 15%). Pathologic T stage was assessed with reference to the American Joint Committee on Cancer (AJCC) eighth edition Staging Manual.

### Body composition parameters from PET/CT imaging

**Figure 2:**
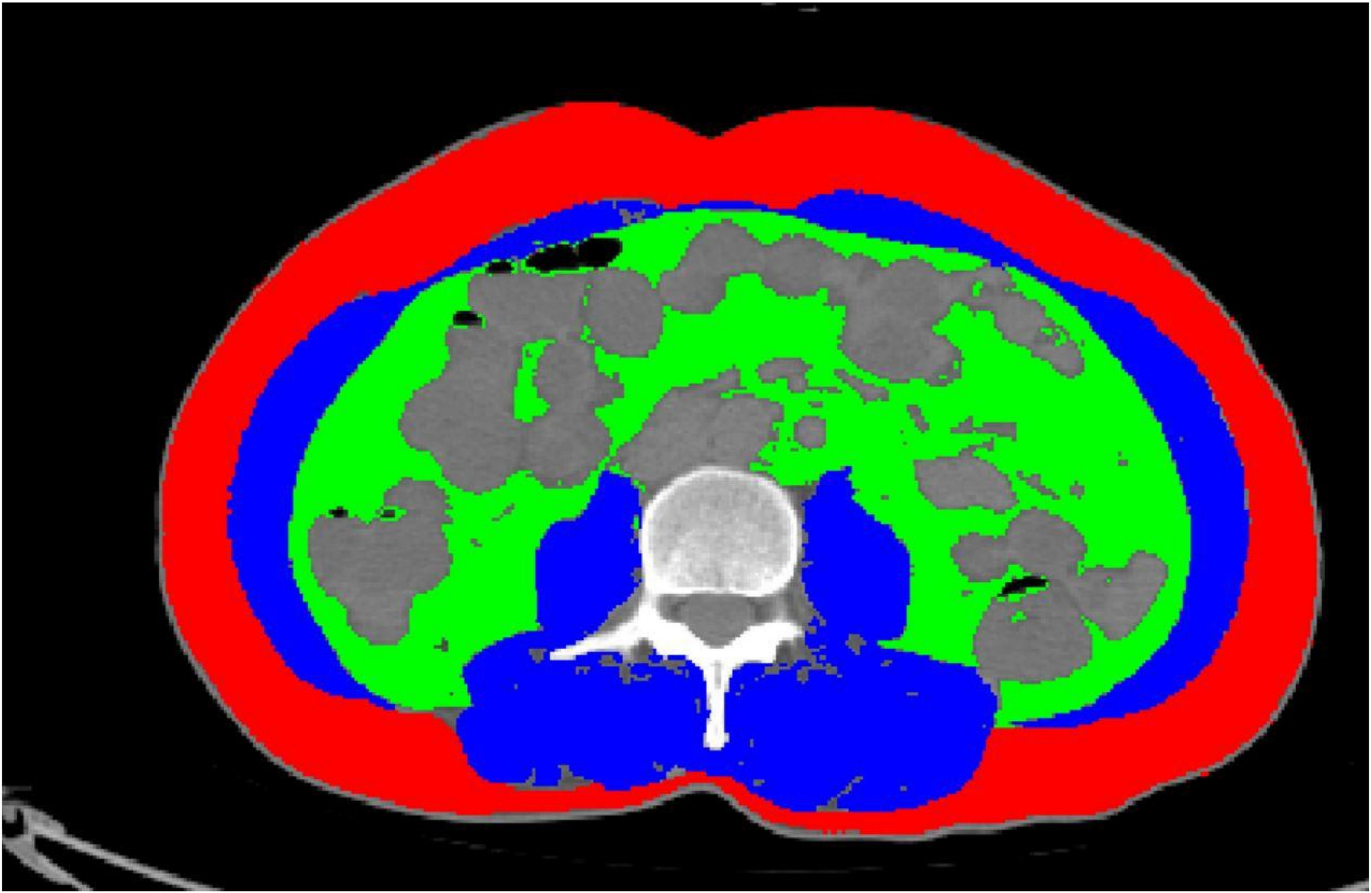
Schematic diagram of VAT, SAT, and SM measurements. Green represents VAT. Red represents SAT. Blue represents SM.

Two experienced nuclear medicine physicians used a commercially available workstation (Xingxiang Technology Information Management System V1.0) to evaluate all images. SAT, VAT, and skeletal muscle (SM) were identified in CT images using anatomic knowledge and predefined tissue-specific Hounsfield units (SAT range: − 30 to − 190, VAT range: − 50 to − 150, SM range: − 29 to 150), as previously described (**Figure 2**) (11). SUV was applied to assess metabolic activity in SAT, VAT, and SM at the level of the third lumbar vertebra (L3). The L3 level was chosen as the SAT metabolic assessment plane because it is widely used in body composition analysis (e.g., fat area measurements, skeletal muscle index) and demonstrates good reproducibility and cross-study comparability (11–14). Additionally, this level is located in the middle of the lumbar spine, and the fat of this level has a stronger correlation with whole-body fat (15, 16). It can stably reflect body fat metabolism status and avoid interference from the downward extension of breast tissue, making it suitable for PET/CT evaluation of breast cancer patients. SUVmax and SUVmean were determined as the highest SUV and the average SUV, respectively.

### Computed tomography body composition analysis

Nonenhanced contrast CT images obtained from PET/CT examinations were analyzed at the L3 level using Slice-O-Matic software (version 5.0; TomoVision, Magog, Canada). Two experienced nuclear medicine physicians measured the cross-sectional areas of the tissues (SAT, VAT, and SM) in square centimeters (cm^2^) and reported them as subcutaneous fat area (SFA), visceral fat area (VFA), and SM area (SMA). These values were normalized to height in square meters (m^2^) and expressed as the SAT index (SATI), VAT index (VATI), and SM index (SMI) in units of square centimeters per square meter (cm^2^/m^2^).

### Statistical analysis

All statistical analyses in this study were performed using R for Windows, version 4.3.1 Patched (https://cran.r-project.org/bin/windows/base/old/4.3.1/). *p*-values < 0.05 were considered statistically significant. Continuous variables were expressed as mean ± SD or median (25th–75th percentile), depending on the distribution of the data. Categorical variables were expressed as frequencies (rates), and comparisons were performed using the Chi-square test. Patient group characteristics were compared using the Chi-square test for categorical variables, the Student’s *t*-test for normally distributed continuous variables, and the Wilcoxon signed-rank test for nonnormally distributed continuous variables. Spearman correlations (*r_s_*) were calculated among the 29 variables included in the analysis.

Variable selection in Cox proportional hazards regression was performed using the least absolute shrinkage and selection operator (LASSO), and the results were verified using the classical random forest algorithm. Specifically, an optimal value for the penalty parameter (λ1) was determined through 1,000-fold internal cross-validation to select variables for the final model. Differences in progression-free survival between groups were assessed using the Kaplan–Meier method and the log-rank test (17). Hazard ratios (HRs) for the associations of the two candidate variables with PFS were estimated in separate Cox regression models. The proportional hazards assumption was tested using the Schoenfeld residuals method, and the nonlinearity assumption for the continuous SUVmean_SAT variable was assessed against the martingale residuals of a null Cox model. To address the nonlinear association of SUVmean_SAT with PFS, SUVmean_SAT was transformed into a classification.

The time-dependent area under the receiver operating characteristic curve (AUC) and the net reclassification index (NRI) (18) were calculated using five different estimators from two models. Additionally, we applied the change-in-effect estimate method (19) to assess confounding and to present the HRs for the association between AJCC stage and PFS after including SUVmean_SAT.

## Results

### Participants characteristics

**Figure 3:**
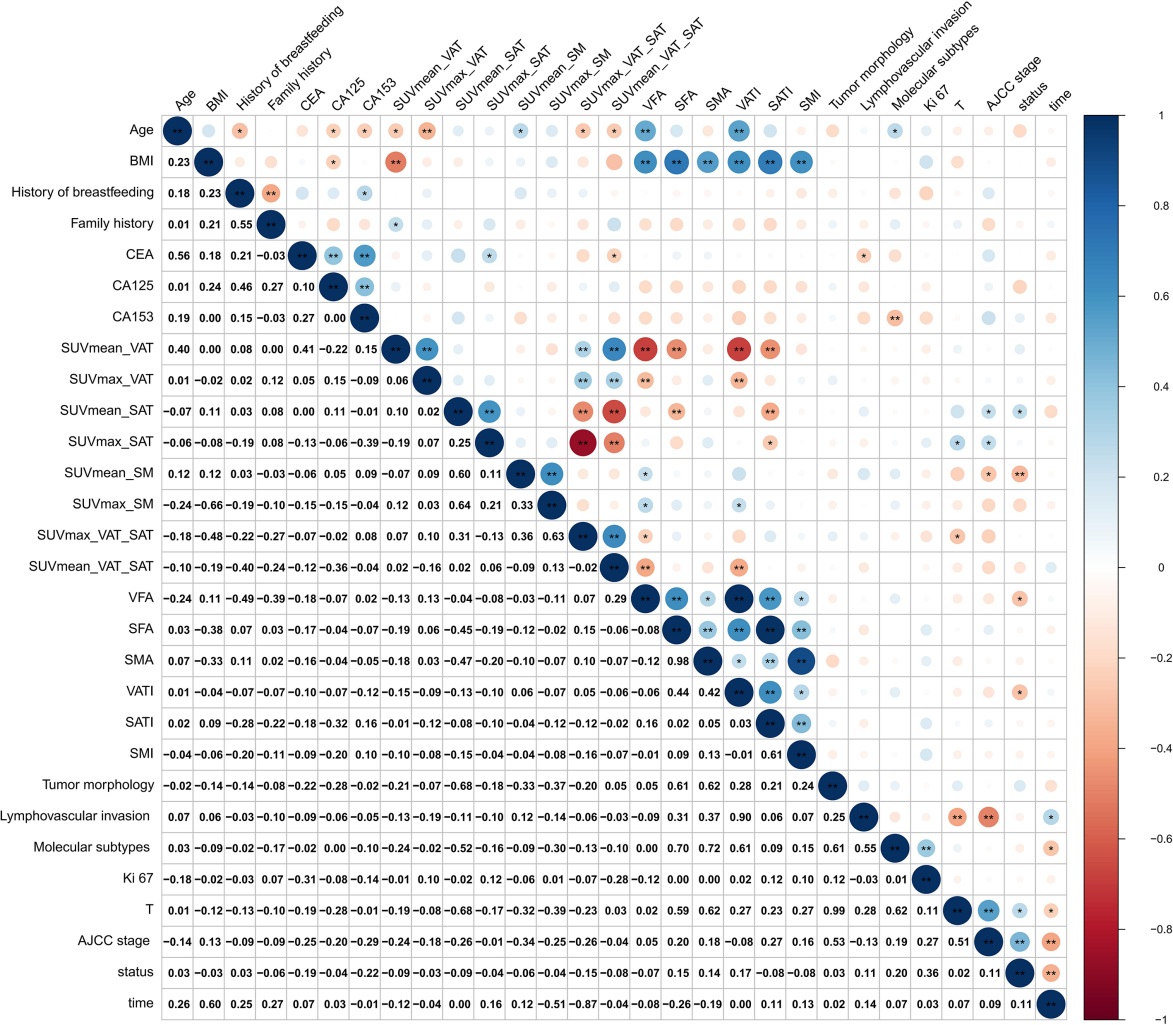
Graphical display of the Pearson correlation matrix of the breast cancer dataset. *p-values < 0.05; **p-values < 0.01.

There were no missing data for continuous covariates or categorical covariates in the study (**Supplementary Figure S1**). The median age at ^18^F-FDG PET/CT scan was 51.5 years (interquartile: 44.3–60.8) among the 74 women included. The majority of patients were stages II–IV (69 [93.2%]). Over a median follow-up of 18.0 months (interquartile range: 10.3–30.8 months), 25 (33.8%) patients experienced disease progression, and 49 (66.2%) remained progression-free (**Table 1**; **Supplementary Figure S2**). SMA was positively correlated with SFA (*r_s_* = 0.98), and molecular subtypes were correlated with SMA (*r_s_* = 0.72) and SFA (*r_s_* = 0.70), respectively. Lymphovascular invasion was highly correlated with VATI (*r_s_* = 0.90), tumor morphology, and pathologic T stage (*r_s_* = 0.99) (**Figure 3**). Further details are presented in **Supplementary File 1**.

**Table 1:** Characteristics of 74 patients with breast cancer included in the study.

Characteristics^a^	Progression-free (*N* = 49)	Progression (*N* = 25)	*p-*value^b^
Age (year; mean ± SD)	53.7 ± 10.8	48.5 ± 12.0	0.064
BMI (kg/m^2^; mean ± SD)	22.6 ± 3.5	22.3 ± 3.8	0.759
History of breastfeeding (*n*; %)			
Yes	47 (95.9%)	24 (96%)	> 0.999
No	2 (4.1%)	1 (4%)	
Family history (*n*; %)			
Yes	1 (2%)	1 (4%)	> 0.999
No	48 (98%)	24 (96%)	
CEA (µg/L; mean ± SD)	10.6 ± 10.6	43.4 ± 126.8	0.209
CA125 (U/ml; median [IQR])	47.7 [14.8, 47.7]	14.5 [12.7, 47.7]	0.065
CA153 (U/ml; mean ± SD)	44.0 ± 92.1	52.8 ± 60.7	0.623
SUVmean_VAT (mean ± SD)	0.5 ± 0.2	0.6 ± 0.2	0.242
SUVmax_VAT (mean ± SD)	0.9 ± 0.2	1.0 ± 0.3	0.355
SUVmean_SAT (median [IQR])	0.21 [0.18, 0.25]	0.26 [0.19, 0.39]	0.037
SUVmax_SAT (mean ± SD)	0.5 ± 0.2	0.5 ± 0.2	0.443
SUVmean_SM (median [IQR])	0.66 [0.60, 0.71]	0.57 [0.53, 0.64]	0.006
SUVmax_SM (mean ± SD)	1.1 ± 0.2	1.0 ± 0.2	0.148
SUVmax_VAT/SAT (mean ± SD)	2.1 ± 0.7	2.2 ± 1.2	0.628
SUVmean_VAT/SAT (mean ± SD)	2.6 ± 0.9	2.4 ± 1.3	0.517
VFA (cm^2^; median [IQR])	94.4 [50.1, 127.5]	49.3 [38.3, 72.0]	0.015
SFA (cm^2^; mean ± SD)	142.9 ± 56.9	131.1 ± 58.0	0.404
SMA (cm^2^; mean ± SD)	99.1 ± 16.6	96.5 ± 16.4	0.532
VATI (cm^2^/m^2^; mean ± SD)	38.6 ± 23.1	25.7 ± 18.6	0.016
SATI (cm^2^/m^2^; mean ± SD)	58.1 ± 23.2	53.3 ± 24.5	0.420
SMI (cm^2^/m^2^; mean ± SD)	40.2 ± 6.9	38.7 ± 6.4	0.376
Tumor morphology			
Mass/Nodular	43 (87.8%)	19 (76%)	0.335
Diffuse	6 (12.2%)	6 (24%)	
Lymphovascular invasion (*n*; %)			
Yes	34 (69.4%)	19 (76%)	0.746
No	15 (30.6%)	6 (24%)	
Molecular subtypes (*n*; %)			
Luminal A^c^	2 (4.1%)	2 (8%)	0.796
Luminal B^d^ [HER2−/HER2+]	25 (51%)	14 (56%)	
HER-2^e^ enriched	14 (28.6%)	5 (20%)	
Triple-negative^f^	8 (16.3%)	4 (16%)	
Ki-67 (*n*; %)			
Low	3 (6.1%)	2 (8%)	> 0.999
High	46 (93.9%)	23 (92%)	
T category (*n*; %)			
T1	10 (20.4%)	3 (12%)	0.095
T2	23 (46.9%)	7 (28%)	
T3	6 (12.2%)	3 (12%)	
T4	10 (20.4%)	12 (48%)	
N category (*n*; %)			
0	14 (28.6%)	4 (16.0%)	0.178
1	15 (30.6%)	4 (16.0%)	
2	11 (22.4%)	9 (36.0%)	
≥ 3	9 (18.4%)	8 (32.0%)	
M category (*n*; %)			
No	38 (77.6%)	8 (32.0%)	< 0.001
Yes	11 (22.4%)	17 (68.0%)	
AJCC stage (*n*; %)			
I	5 (10.2%)	0 (0%)	0.001
II	19 (38.8%)	3 (12%)	
III	14 (28.6%)	5 (20%)	
IV	11 (22.4%)	17 (68%)	
Follow-up time (months; median [IQR])	22.0 [12.0, 36.0]	12.0 [6.0, 21.0]	0.002

*SD*, standard deviation; *IQR*, interquartile range.

a Continuous variables are presented as mean ± SD for normally distributed data or median (IQR) for nonnormally distributed data, and categorical variables as number (%).

b *p*-values were calculated using the Student’s *t*-test for normally distributed data or the Wilcoxon signed-rank test for nonnormally distributed continuous variables, and the *χ*^2^ test for categorical variables.

c High ESR1, PGR, BCL2; low proliferation (e.g., MKI67), estrogen receptor-positive, best prognosis.

d ER/PR+, but with elevated proliferation signatures (MKI67, CCNB1), intermediate prognosis; more likely to benefit from chemotherapy.

e High ERBB2-pathway gene expression, often, but not exclusively, HER2 amplification; benefits from anti-HER2 therapy.

f Lacking ER, PR, HER2; enriched for proliferation and basal cytokeratin genes (KRT5/14/17), poor prognosis.

### Independent predictors for PFS of breast cancer

**Figure 4:**
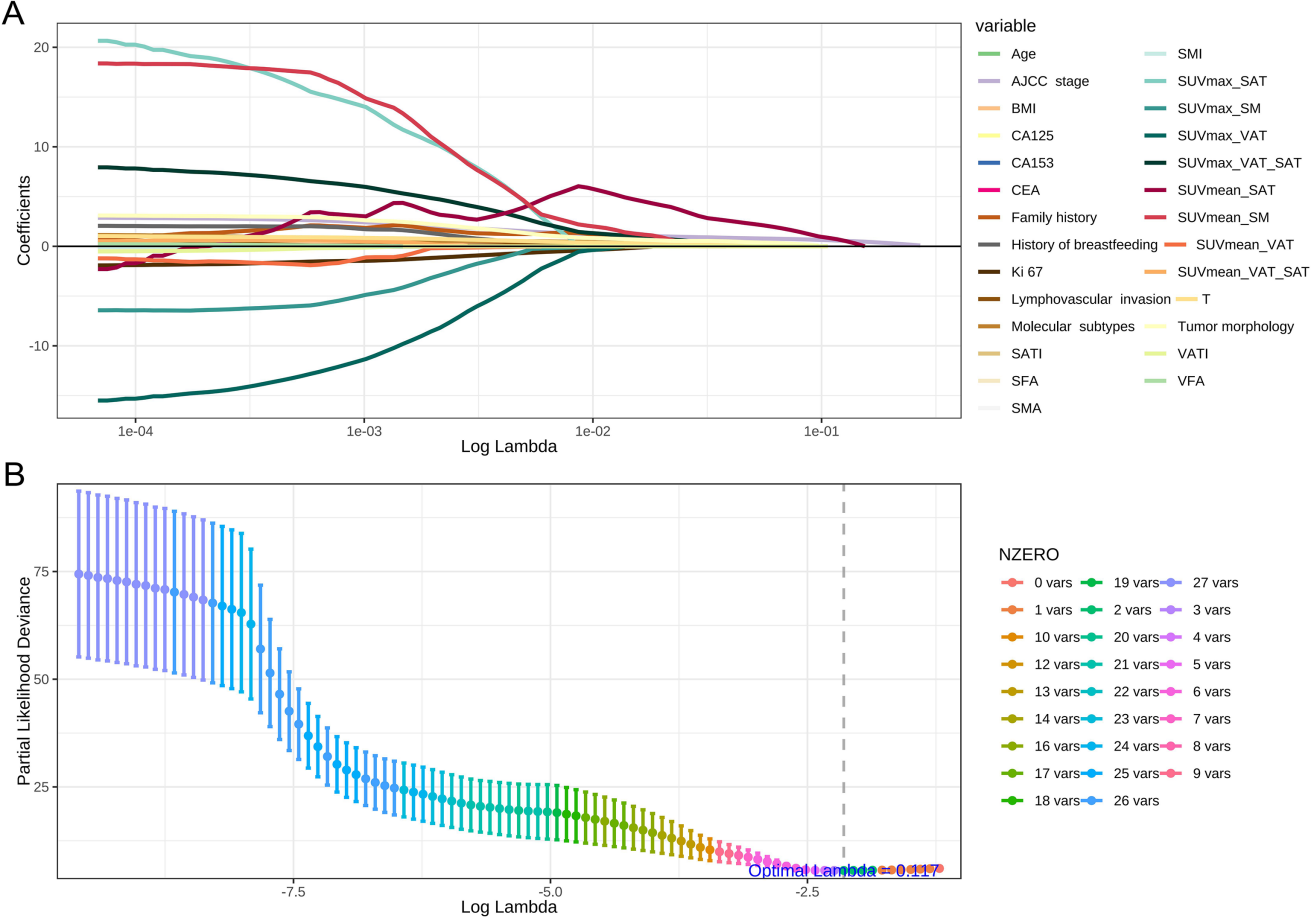
LASSO Cox regression for variable selection. **(A)** LASSO coefficient profiles of the 27 candidate risk factors in the training set. **(B)** Two risk factors were selected using LASSO Cox regression analysis. The vertical dashed line was drawn at the optimal score of 0.117 on the minimum criteria.

Patients with high SUVmean_SAT or low SUVmean_SM had lower PFS (*p* < 0.05). Patients with low VFA and VATI also had lower PFS (*p* < 0.05). A LASSO-penalized multivariate survival model was constructed to reduce model overfitting during the selection of risk indicators using the glmnet R Package (20). To further identify the most promising clinical factors, LASSO Cox modeling was performed with 1,000-fold cross-validation. Across 1,000 iterations, two nonzero coefficient variables (coefficient: AJCC stage = 0.71, SUVmena_SAT = 0.58) were selected for further analysis, with the optimal lambda value (0.117) that minimized the error (**Figure 4**) (21).

Two Cox models were developed from the candidate variables: model 1 included AJCC stage, and model 2 included AJCC stage plus SUVmean_SAT. The proportional hazards assumption was checked using statistical tests and graphical diagnostics based on the scaled Schoenfeld residuals, and no violation was detected, as shown in **Supplementary Figure S3**. However, the continuous covariate SUVmean_SAT displayed nonlinearity (**Supplementary Figure S4**). The significant cutoff value for the SUVmean_SAT continuous variable in the Cox regression was calculated, and patients were divided into high and low SUVmean_SAT groups based on the optimal cutoff value of 0.27 (**Supplementary Table S1**). Additionally, **Supplementary Figure S5** shows that random forests for survival analysis were employed to validate the LASSO findings, and the computation of variable importance rankings yielded consistent results for the two variables (AJCC stage, SUVmean_SAT).

### Cox model construction and evaluation

**Figure 5:**
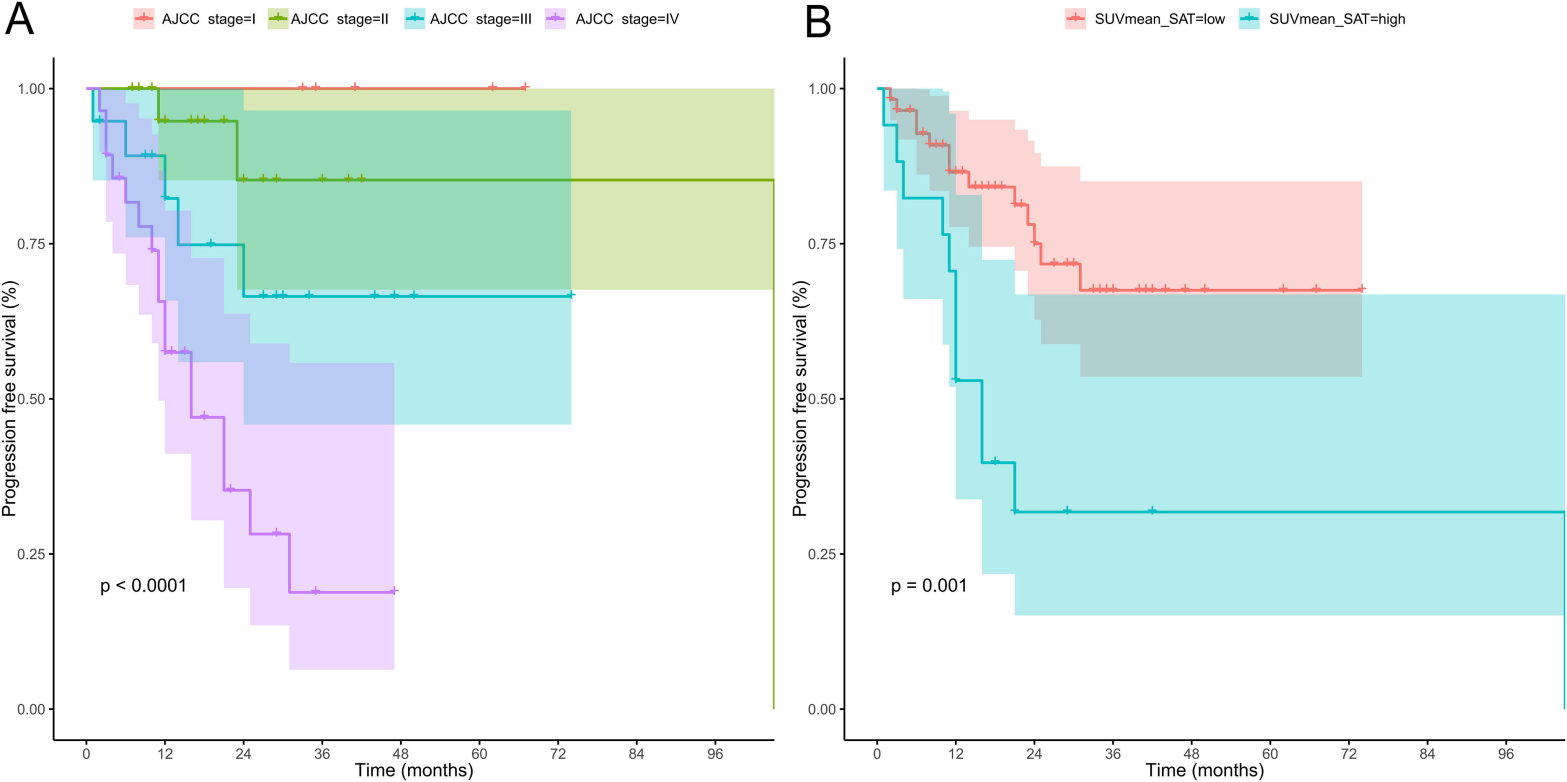
The Kaplan–Meier survival curves for the two candidate variables. **(A)** Displayed four groups by AJCC stage (I to IV), showing lower survival for higher stages. **(B)** Compared low versus high SAT SUVmean, with high SAT SUVmean associated with lower progression-free survival. Both **(A)** and **(B)** includeshaded confidence intervals and statistically significant p-values.

The Kaplan–Meier survival curves and log-rank test results for the two candidate variables are shown in **Figure 5**. The results indicated that high AJCC stage (*p* < 0.0001) and high SUVmean_SAT (*p* = 0.001) were significantly associated with shorter PFS. Cox regression analysis showed that AJCC stage (HR from model 2: 2.960 [1.605–5.458]) and SUVmean_SAT (HR from model 2: 2.279 [0.971–5.350]) were independent risk factors for prognosis in breast cancer patients (**Supplementary Table S2**).

**Figure 6:**
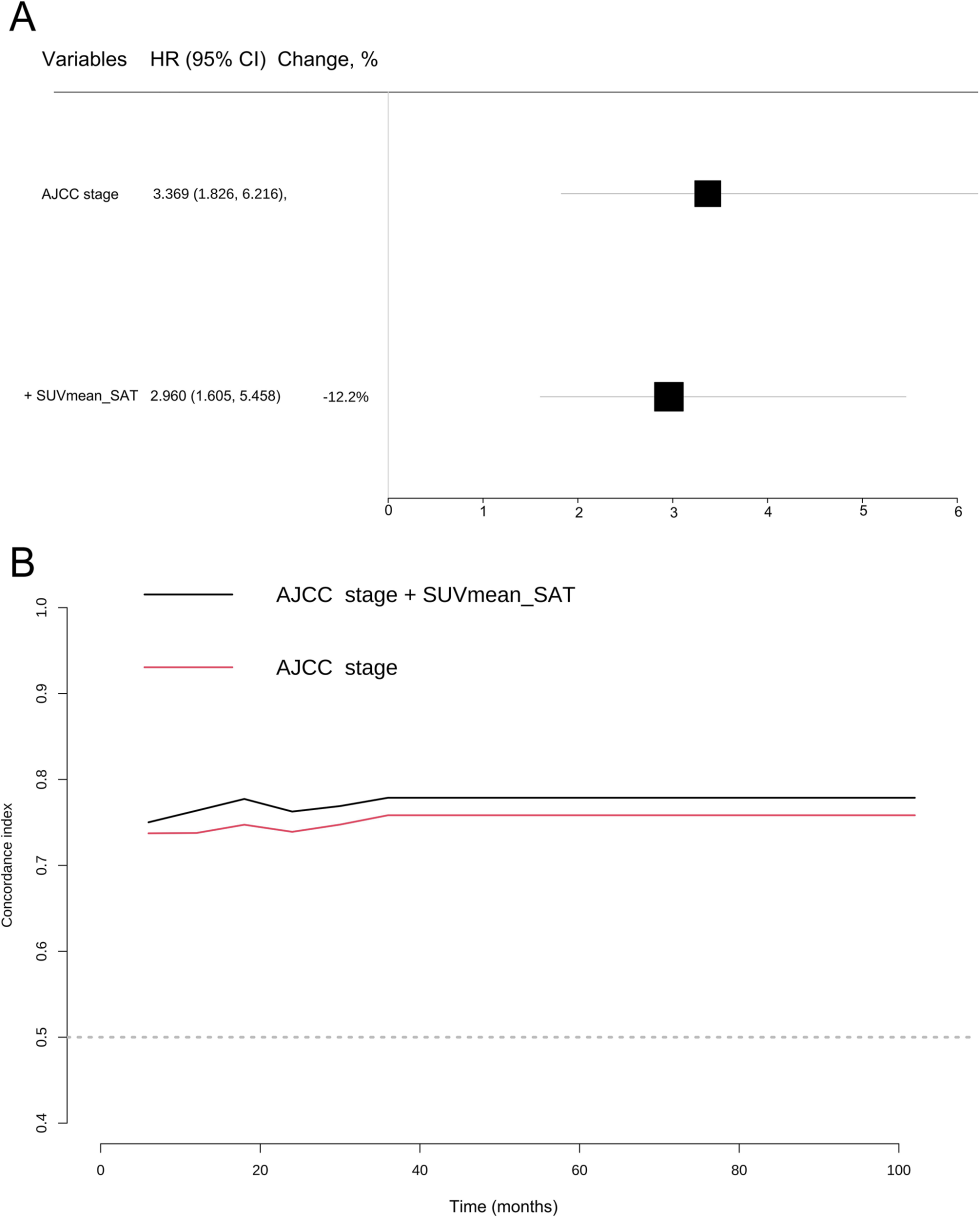
**(A)** Assessment of confounding effects in the association between AJCC stage and progression-free outcome by adding the potential confounders SUVmean_SAT to the model. **(B)** Time-dependent concordance index for the breast cancer dataset.

The time-dependent AUC values of model 2 were higher than those of model 1 at 12, 24, 36, and 60 months (**Table 2**; **Figure 6B**). We also calculated the NRI for the two Cox models using up to five different estimators, including the Kaplan–Meier estimator, inverse probability weighted estimator, smooth inverse probability weighted estimator, semiparametric estimator, and combined estimator, as described (along with all other estimates) (**Table 3**). The NRI values at 12, 24, 36, and 60 months (combined method: 0.608, 0.392, 0.347, 0.347) were all greater than 0.3, indicating an improvement in prediction performance gained by adding the SUVmean_SAT to the AJCC stage (22). The change in effect estimate showed a 12.2% decrease in the hazard ratios (HR of AJCC stage in model 2: 2.960 [1.605–5.458]) when SUVmean_SAT was added to the individual AJCC stage model (HR of AJCC stage in model 1: 3.369 [1.826–6.216]) (**Figure 6A**; **Supplementary Table S2**).

**Table 2:** Comparison of time-dependent ROC analyses for the two models.

AUC	12 months	24 months	36 months	60 months
Model 1: AJCC stage	0.738	0.739	0.758	0.758
	0.764	0.763	0.779	0.779

*AUC*, area under the curve.

**Table 3:** Net reclassification index of the two Cox models.

Methods	12 months	24 months	36 months	60 months
KW	0.661	0.384	0.228	0.228
IPW	0.826	0.623	0.460	0.460
SmoothIPW	0.697	0.436	0.260	0.260
SEM	0.602	0.389	0.352	0.352
Combined	0.608	0.392	0.347	0.347

*KM*, Kaplan–Meier estimator; *IPW*, inverse probability weighted estimator; *SmoothIPW*, smooth inverse probability weighted estimator; *SEM*, semiparametric estimator; *Combined*, combined estimator as described (along with all other estimates).

## Discussion

### Glucose metabolic activity of SAT and PFS of breast cancer

In this study, we found that higher glucose metabolic activity of SAT was significantly associated with increased risk of PFS events among patients with breast cancer. To the best of our knowledge, this is the first study to investigate the glucose metabolic activity of adipose tissue in relation to PFS in Chinese breast cancer patients.

At present, most relevant studies have examined the relationship between CT parameters (radioactivity, area, or volume) of adipose tissue and the prognosis of breast cancer (23–28). The primary outcome of these studies was PFS or mortality, but the best predictors reported varied among SAT, VAT, and the ratio of VAT to SAT. In American patients with nonmetastatic breast cancer (*n* = 2,868), high radioactivity of SAT (rather than VAT) was associated with an increased overall risk of mortality (HR: 1.45 [1.15–1.81]) (25). In Canadian patients with nonmetastatic breast cancer (*n* = 3,235), an increase in SFA was associated with mortality in stage II/III breast cancer (HR: 1.13 [1.02–1.26]) (27). Both of the studies with the largest sample sizes suggested that SAT was a risk factor for breast cancer mortality; however, they did not include metabolic parameters of adipose tissue.

To date, only three studies have investigated the associations of glucose metabolic activity of adipose tissue and metastasis or outcomes in breast cancer (7–9). Only one study reported PFS as an outcome and suggested that high SUVmean_VAT was associated with poor recurrence-free survival (HR: 2.754 [1.090–6.958]) (8). This finding is not consistent with the results of the present study, which indicated that high SUVmean_SAT was associated with poor recurrence-free survival (HR: 2.279 [0.971–5.350]). A possible explanation is that, since most breast tissue is adipose tissue, abdominal SAT can capture whole-body changes, including those in breasts, and provide additional information for predicting breast cancer prognosis after diagnosis (29). Another explanation is that high SUVmean_SAT may reflect a stronger inflammatory response (based on imaging phenotypes rather than direct experimental validation) (30), which is a key component of tumor progression (31). Similar findings have been observed in other tumor studies; SAT is also a prognostic factor for gastric cancer (32), metastatic colorectal cancer (33), and liver cancer (34).

### Prediction model of SAT and AJCC stage

The traditional AJCC staging of breast cancer is based on the anatomic stage, including tumor size (T), regional lymph node metastasis (N), and distant metastasis (M) (35). It is estimated that 6%–10% of breast cancer patients are at AJCC stage IV at the time of diagnosis, and these patients have a poor prognosis, with a median survival time of 2–3 years (36, 37). So, the AJCC stage is a widely accepted tool for predicting the prognosis of patients. However, the factors included in the AJCC stage are limited, making it difficult to accurately predict individual breast cancer outcomes using this tool (38). This study’s findings suggest that AJCC stage and SUVmean_SAT are independent risk factors for prognosis in patients with breast cancer, even more significant than pathologic parameters such as molecular subtypes, pathologic T stage, and Ki-67.

Therefore, we compared the individual AJCC stage model with the combined model of SUVmean_SAT plus AJCC stage. The results showed that the prediction efficiency of the combined model was higher than that of the individual AJCC model (NRI > 0.3), indicating that SUVmean_SAT provides additional information beyond AJCC stage for predicting breast cancer prognosis.

### Strengths and weaknesses

One of the strengths of this study is that the data collected included clinical history, serum bio-markers, pathological parameters, CT parameters, and metabolic activity parameters. Another strength is that the study calculated the NRI of the Cox regression models using up to five different estimators and further assessed the potential confounding effect in the association between SUVmean_SAT, AJCC stage, and progression status in breast cancer patients.

This study has several limitations. First, the relatively small sample size, particularly the limited number of progression-free survival events, means that the current results should be regarded as preliminary and primarily serve for hypothesis generation. Future external validation in larger, multicenter cohorts is required. Second, this study only included Chinese participants, and significant differences exist in body fat distribution and the prevalence of metabolic syndrome across different races. Validation in larger, multiethnic cohorts is needed. Third, with a median follow-up time of 18 months, the study primarily captures early- to mid-term disease progression signals and is insufficient to fully reflect long-term recurrence patterns. Extending follow-up in future studies will help further evaluate the predictive value of SAT metabolic activity for late recurrence. Finally, breast cancer is a highly heterogeneous disease in histology, epidemiology, and molecular characteristics. This study did not compare whether the prognosis value of SUVmean_SAT differs across molecular subtypes. Whether SUVmean_SAT consistently predicts outcomes across different subtypes remains to be verified in larger-sample studies.

## Conclusion

High glucose metabolic activity in SAT and AJCC stage are independent risk factors for PFS in patients with breast cancer. The combined prediction model of SUVmean_SAT and AJCC stage can more accurately evaluate breast cancer progression. This study may deepen the understanding of the influence of adiposity on breast cancer prognosis.

## Data Availability

The raw data supporting the conclusions of this article will be made available by the authors, without undue reservation.
