# Supplementary material for: Factors associated with clinical outcomes of breast cancer based on glucose metabolic activity of subcutaneous adipose tissue
Source: Front Oncol. 2026 Feb 23;16:1722085. doi: 10.3389/fonc.2026.1722085 (PMC12967984; doi:10.3389/fonc.2026.1722085)

DATA DIAGNOSIS REPORT
BC

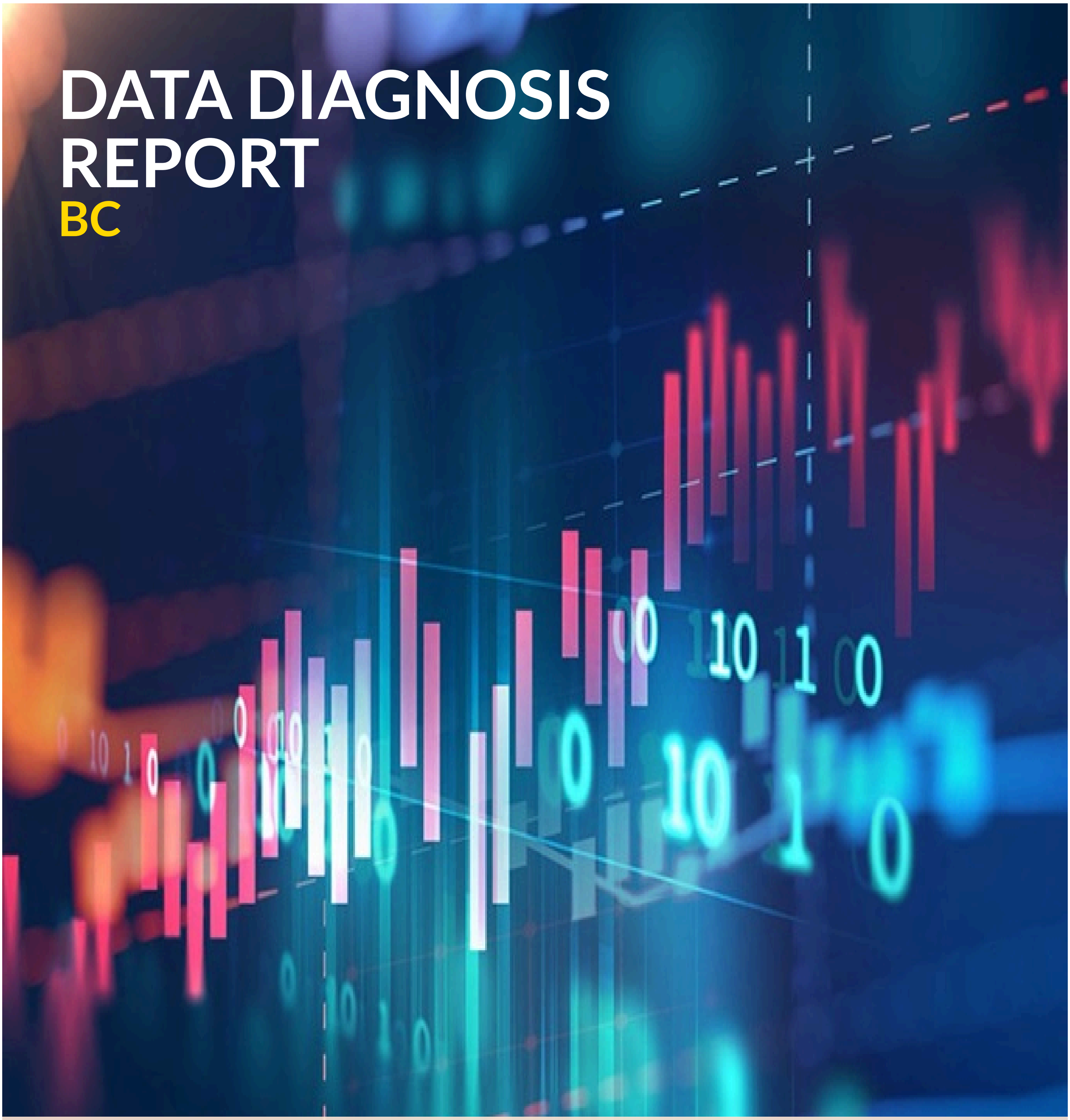

Report Overview

This report was created for an overview quality diagnosis of ***bc*** data. It was created for **the purpose of judging the validity of variables** before conducting EDA.

Contents

Overview 2
- Data Structures 2
- Job Informations 2
- Warnings 3
- Variables 5

Missing Values 7
- List of Missing Values 7
- Visualization 7

Unique Values 8
- Categorical Vaiables 8
- Numerical Vaiables 9

Categorical Variable Diagnosis 10
- Top Ranks 10

Numerical Variable Diagnosis 11
- Distributions 11
  - Zero Values 13
  - Negative Values 14
- Outliers 15
  - List of Outliers 15
  - Individual Outliers 16

Overview

Data Structures

division	metrics	value
size	observations	74
size	variables	29
size	values	2,146
size	memory size (KB)	0
duplicated	duplicate observation	0
missing	complete observation	74
missing	missing observation	0
missing	missing variables	0
missing	missing values	0

division	metrics	value
data type	numerics	18
data type	integers	11
data type	factors/ordered	0
data type	characters	0
data type	Dates	0
data type	POSIXcts	0
data type	others	0

Table 1: Data structures and types

Job Informations

division	metrics	value
dataset	dataset	bc
dataset	dataset type	data.frame
job	samples	74 / 74 (100%)
job	created	2024-04-23 08:04:41.262846
job	created by	dlookr

Table 2: Job informations

Warnings

checks	judgements	removes
5	30	0

Table 3: Summary of warnings

warnings	status	recommend
VFA has high(1.00) cardinality, Maybe identifier	cardinality	check
VATI has high(1.00) cardinality, Maybe identifier	cardinality	check
SATI has high(1.00) cardinality, Maybe identifier	cardinality	check
SMI has high(1.00) cardinality, Maybe identifier	cardinality	check
History.of.breastfeeding has a low cardinality. 2 (2.7%) distinct values	cardinality	judgement
Family.history has a low cardinality. 2 (2.7%) distinct values	cardinality	judgement
Tumor.morphology has a low cardinality. 2 (2.7%) distinct values	cardinality	judgement
Lymphovascular_invasion has a low cardinality. 2 (2.7%) distinct values	cardinality	judgement
Molecular_subtypes has a low cardinality. 4 (5.4%) distinct values	cardinality	judgement
Ki.67 has a low cardinality. 2 (2.7%) distinct values	cardinality	judgement
T has a low cardinality. 4 (5.4%) distinct values	cardinality	judgement
AJCC_stage has a low cardinality. 4 (5.4%) distinct values	cardinality	judgement
status has a low cardinality. 2 (2.7%) distinct values	cardinality	judgement
status has 49 (66.22%) zeros	zero	check
Tumor.morphology has 12 (16.22%) outliers	outlier	judgement
SUVmean_SAT has 7 (9.46%) outliers	outlier	judgement
CA125 has 5 (6.76%) outliers	outlier	judgement
CA153 has 5 (6.76%) outliers	outlier	judgement
Ki.67 has 5 (6.76%) outliers	outlier	judgement
History.of.breastfeeding has 3 (4.05%) outliers	outlier	judgement
CEA has 3 (4.05%) outliers	outlier	judgement
SUVmax_SM has 3 (4.05%) outliers	outlier	judgement

Table 4: Warnings in dataset and variables

	warnings	status	recommend
	**warnings**	**status**	**recommend**
23	SFA has 3 (4.05%) outliers	outlier	judgement
24	time has 3 (4.05%) outliers	outlier	judgement
25	BMI has 2 (2.7%) outliers	outlier	judgement
26	Family.history has 2 (2.7%) outliers	outlier	judgement
27	SATI has 2 (2.7%) outliers	outlier	judgement
28	SMI has 2 (2.7%) outliers	outlier	judgement
29	SUVmean_VAT has 1 (1.35%) outliers	outlier	judgement
30	SUVmax_VAT has 1 (1.35%) outliers	outlier	judgement
31	SUVmax_SAT has 1 (1.35%) outliers	outlier	judgement
32	SUVmean_SM has 1 (1.35%) outliers	outlier	judgement
33	SUVmax_VAT_SAT has 1 (1.35%) outliers	outlier	judgement
34	VFA has 1 (1.35%) outliers	outlier	judgement
35	VATI has 1 (1.35%) outliers	outlier	judgement

Table 4: Warnings in dataset and variables (continued)

Variables

variables	types	missing	cardinality	zero	minus	outlier
Age	integer					
BMI	numeric					X
History.of.breastfeeding	integer		< low			X
Family.history	integer		< low			X
CEA	numeric					X
CA125	numeric					X
CA153	numeric					X
SUVmean_VAT	numeric					X
SUVmax_VAT	numeric					X
SUVmean_SAT	numeric					X
SUVmax_SAT	numeric					X
SUVmean_SM	numeric					X
SUVmax_SM	numeric					X
SUVmax_VAT_SAT	numeric					X
SUVmean_VAT_SAT	numeric					
VFA	numeric		identifier			X
SFA	numeric					X
SMA	numeric					
VATI	numeric		identifier			X
SATI	numeric		identifier			X
SMI	numeric		identifier			X
Tumor.morphology	integer		< low			X
Lymphovascular_invasion	integer		< low			
Molecular_subtypes	integer		< low			
Ki.67	integer		< low			X

Table 5: List of variables diagnosis

variables	types	missing	cardinality	zero	minus	outlier
**variables**	**types**	**missing**	**cardinality**	**zero**	**minus**	**outlier**
T	integer		< low			
AJCC_stage	integer		< low			
status	integer		< low	X		
time	integer					X

Table 5: List of variables diagnosis (continued)

Missing Values

List of Missing Values

No variables including missing values

Visualization

No variables including missing values

Unique Values

Categorical Vaiables

No variable with a high proportion greater than 0.5

Numerical Vaiables

Variables where the unique cases is less than 5 or unique is 1.

variables	types	unique	unique (%)	status	recommend
History.of.breastfeeding	integer	2	2.7%	low cardinality	Judgment
Family.history	integer	2	2.7%	low cardinality	Judgment
Tumor.morphology	integer	2	2.7%	low cardinality	Judgment
Lymphovascular_invasion	integer	2	2.7%	low cardinality	Judgment
Molecular_subtypes	integer	4	5.4%	low cardinality	Judgment
Ki.67	integer	2	2.7%	low cardinality	Judgment
T	integer	4	5.4%	low cardinality	Judgment
AJCC_stage	integer	4	5.4%	low cardinality	Judgment
status	integer	2	2.7%	low cardinality	Judgment

Table 6: Detail warning numerical cardinality

Categorical Variable Diagnosis

Top Ranks

No categorical variable

Numerical Variable Diagnosis

Distributions

variables	min	Q1	mean	median	Q3	max	zero	minus	outlier
Age	27.00	44.25	51.95	51.50	60.75	86.00	0	0	0
BMI	14.00	20.05	22.47	22.00	24.08	34.40	0	0	2
History.of.breastfeeding	1.00	1.00	1.04	1.00	1.00	2.00	0	0	3
Family.history	1.00	2.00	1.97	2.00	2.00	2.00	0	0	2
CEA	0.65	2.17	21.70	4.62	21.73	612.50	0	0	3
CA125	7.26	13.45	48.15	47.72	47.72	378.00	0	0	5
CA153	3.48	17.24	46.96	29.59	46.48	658.80	0	0	5
SUVmean_VAT	0.29	0.43	0.56	0.57	0.67	1.10	0	0	1
SUVmax_VAT	0.54	0.78	0.94	0.96	1.05	1.95	0	0	1
SUVmean_SAT	0.13	0.18	0.25	0.22	0.27	0.63	0	0	7
SUVmax_SAT	0.24	0.35	0.51	0.45	0.62	1.10	0	0	1
SUVmean_SM	0.48	0.56	0.64	0.64	0.70	1.13	0	0	1
SUVmax_SM	0.65	0.92	1.04	1.00	1.12	1.90	0	0	3
SUVmax_VAT_SAT	0.82	1.50	2.14	2.04	2.74	6.29	0	0	1
SUVmean_VAT_SAT	0.69	1.66	2.50	2.39	3.20	5.50	0	0	0
VFA	8.66	43.01	84.44	66.65	120.62	255.80	0	0	1
SFA	14.09	103.85	138.94	133.65	162.65	368.60	0	0	3
SMA	70.86	85.96	98.19	96.58	107.33	139.50	0	0	0
VATI	3.18	17.06	34.21	25.66	46.74	99.92	0	0	1
SATI	5.18	41.39	56.47	53.31	66.37	147.65	0	0	2
SMI	26.03	34.82	39.70	38.85	42.82	59.29	0	0	2
Tumor.morphology	1.00	1.00	1.16	1.00	1.00	2.00	0	0	12
Lymphovascular_invasion	1.00	1.00	1.28	1.00	2.00	2.00	0	0	0
Molecular_subtypes	1.00	2.00	2.53	2.00	3.00	4.00	0	0	0
Ki.67	1.00	2.00	1.93	2.00	2.00	2.00	0	0	5

Table 7: General list of numerical diagnosis

	variables	min	Q1	mean	median	Q3	max	zero	minus	outlier
	variables	min	Q1	mean	median	Q3	max	zero	minus	outlier
26	T	1	2.00	2.54	2	4.00	4	0	0	0
27	AJCC_stage	1	2.00	2.95	3	4.00	4	0	0	0
28	status	0	0.00	0.34	0	1.00	1	49	0	0
29	time	1	10.25	22.80	18	30.75	105	0	0	3

Table 7: General list of numerical diagnosis (continued)

Zero Values

variables	min	median	max	zero	zero (%)
status	0	0	1	49	66.2

Table 8: List of numerical diagnosis (zero)

Negative Values

No numeric variable with negative value

Outliers

List of Outliers

variables	min	median	max	outlier	outlier (%)
Tumor.morphology	1.00	1.00	2.00	12	16.2
SUVmean_SAT	0.13	0.22	0.63	7	9.5
CA125	7.26	47.72	378.00	5	6.8
CA153	3.48	29.59	658.80	5	6.8
Ki.67	1.00	2.00	2.00	5	6.8
History.of.breastfeeding	1.00	1.00	2.00	3	4.1
CEA	0.65	4.62	612.50	3	4.1
SUVmax_SM	0.65	1.00	1.90	3	4.1
SFA	14.09	133.65	368.60	3	4.1
time	1.00	18.00	105.00	3	4.1
BMI	14.00	22.00	34.40	2	2.7
Family.history	1.00	2.00	2.00	2	2.7
SATI	5.18	53.31	147.65	2	2.7
SMI	26.03	38.85	59.29	2	2.7
SUVmean_VAT	0.29	0.57	1.10	1	1.4
SUVmax_VAT	0.54	0.96	1.95	1	1.4
SUVmax_SAT	0.24	0.45	1.10	1	1.4
SUVmean_SM	0.48	0.64	1.13	1	1.4
SUVmax_VAT_SAT	0.82	2.04	6.29	1	1.4
VFA	8.66	66.65	255.80	1	1.4
VATI	3.18	25.66	99.92	1	1.4

Table 9: Diagnosis of numerical variable outliers

Individual Outliers

variable: Tumor.morphology

Measures	Values
Outliers count	12
Outliers ratio (%)	16.22%
Mean of outliers	2
Mean with outliers	1.162162
Mean without outliers	1

Table 10: Tumor.morphology

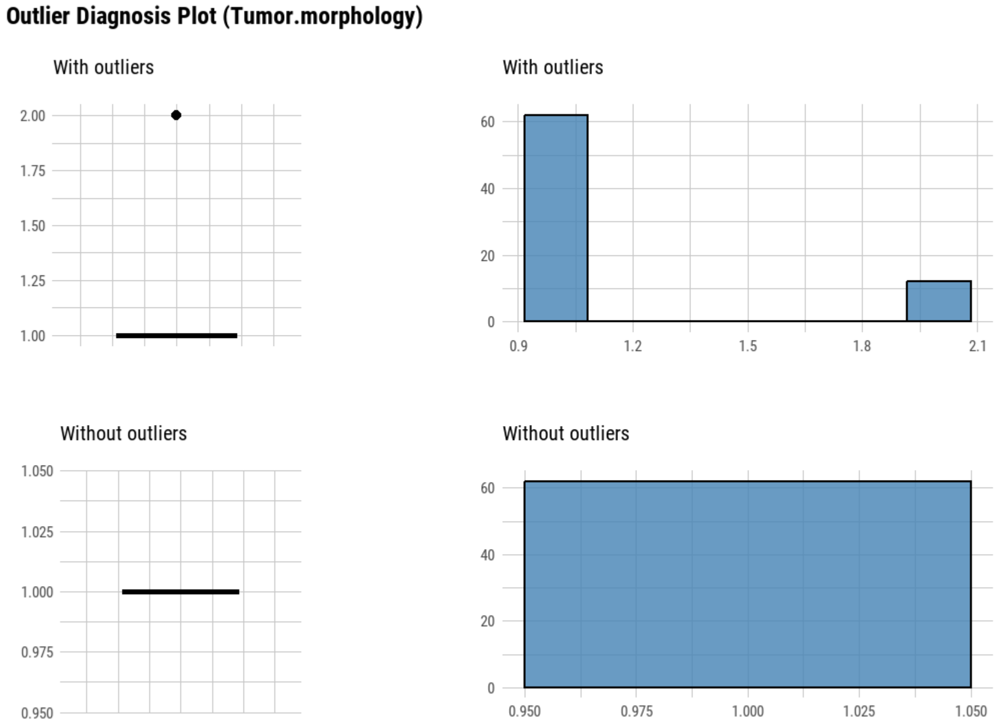

variable: SUVmean_SAT

Measures	Values
Outliers count	7
Outliers ratio (%)	9.46%
Mean of outliers	0.47
Mean with outliers	0.2482432
Mean without outliers	0.2250746

Table 10: SUVmean_SAT

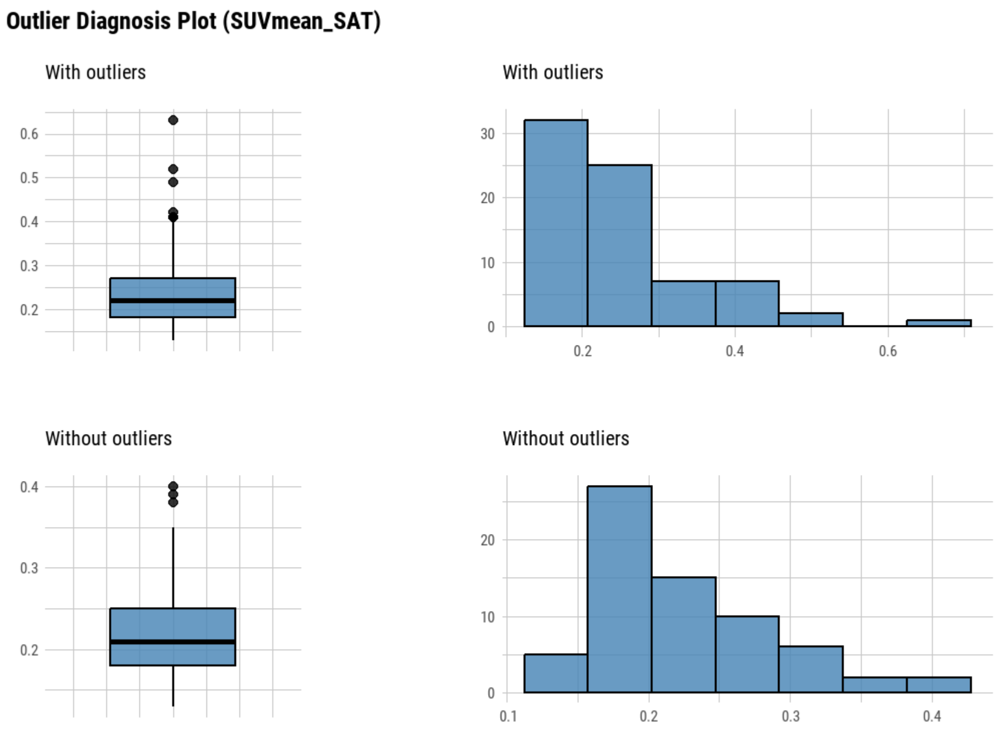

variable: CA125

Measures	Values
Outliers count	5
Outliers ratio (%)	6.76%
Mean of outliers	253.98
Mean with outliers	48.15474
Mean without outliers	33.23986

Table 10: CA125

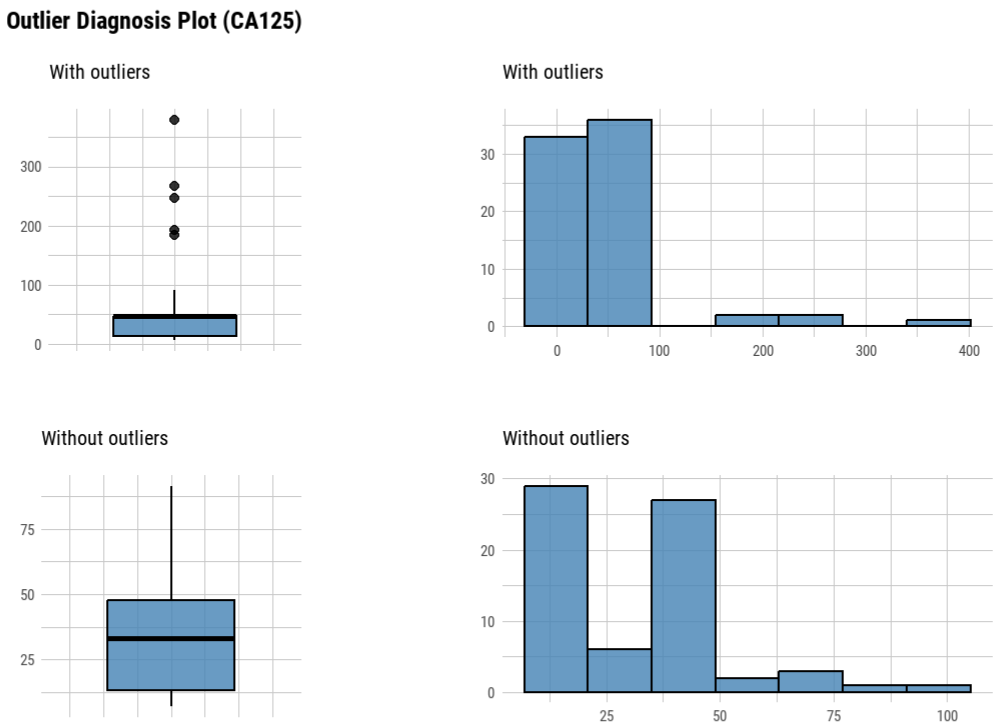

variable: CA153

Measures	Values
Outliers count	5
Outliers ratio (%)	6.76%
Mean of outliers	278.32
Mean with outliers	46.96366
Mean without outliers	30.19871

Table 10: CA153

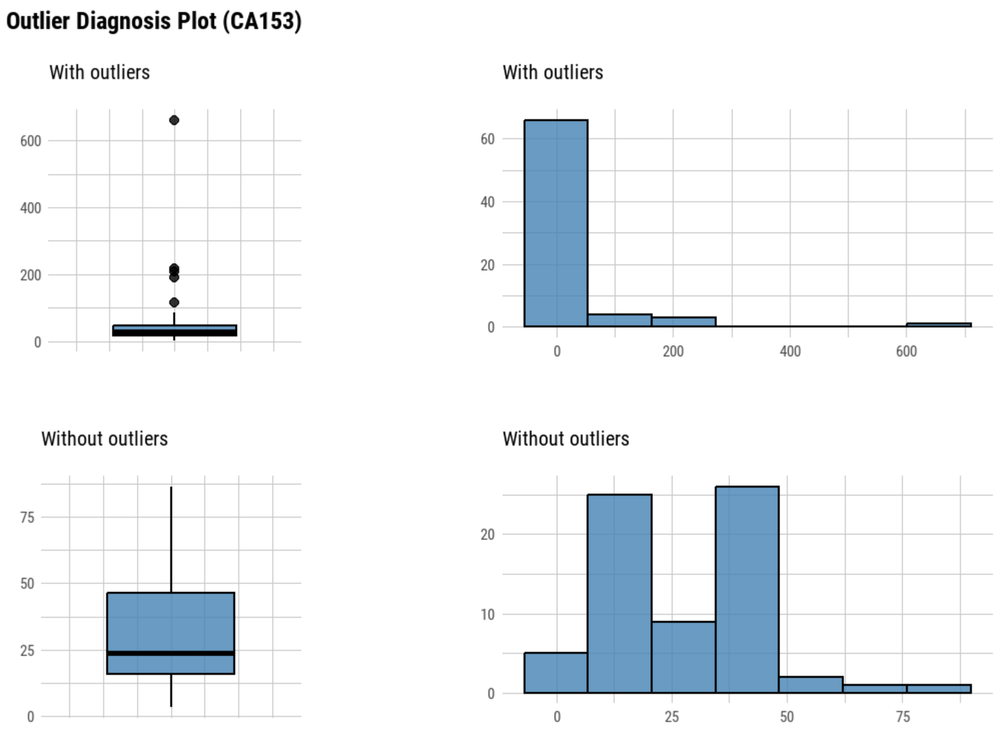

variable: Ki.67

Measures	Values
Outliers count	5
Outliers ratio (%)	6.76%
Mean of outliers	1
Mean with outliers	1.932432
Mean without outliers	2

Table 10: Ki.67

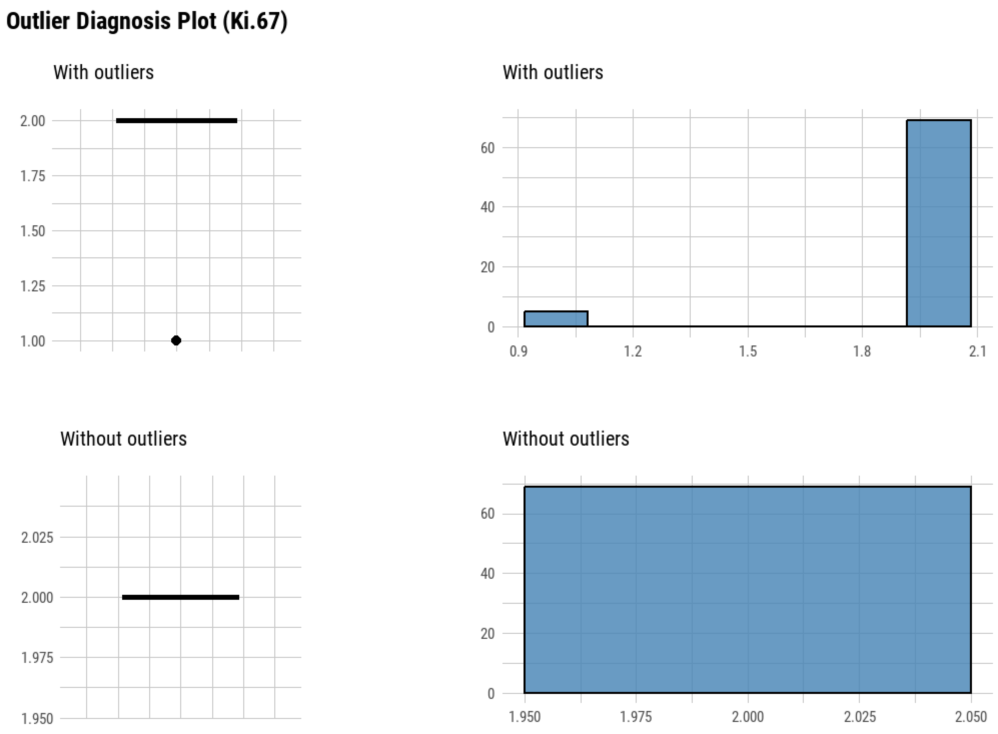

variable: History.of.breastfeeding

Measures	Values
Outliers count	3
Outliers ratio (%)	4.05%
Mean of outliers	2
Mean with outliers	1.040541
Mean without outliers	1

Table 10: History.of.breastfeeding

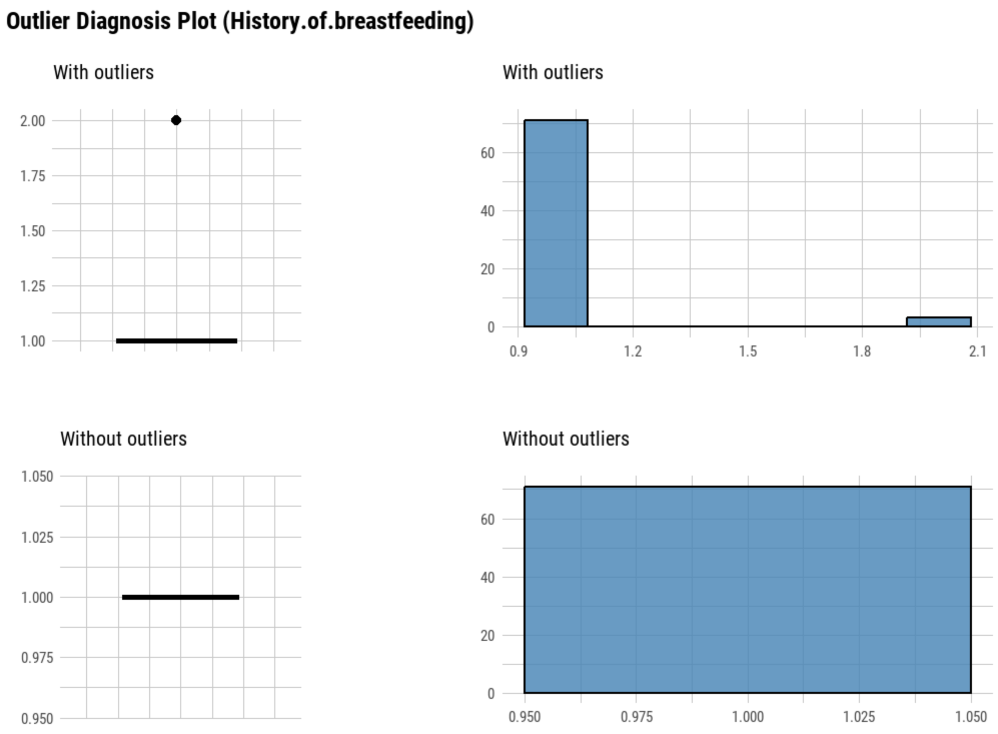

variable: CEA

Measures	Values
Outliers count	3
Outliers ratio (%)	4.05%
Mean of outliers	297.9167
Mean with outliers	21.69688
Mean without outliers	10.02562

Table 10: CEA

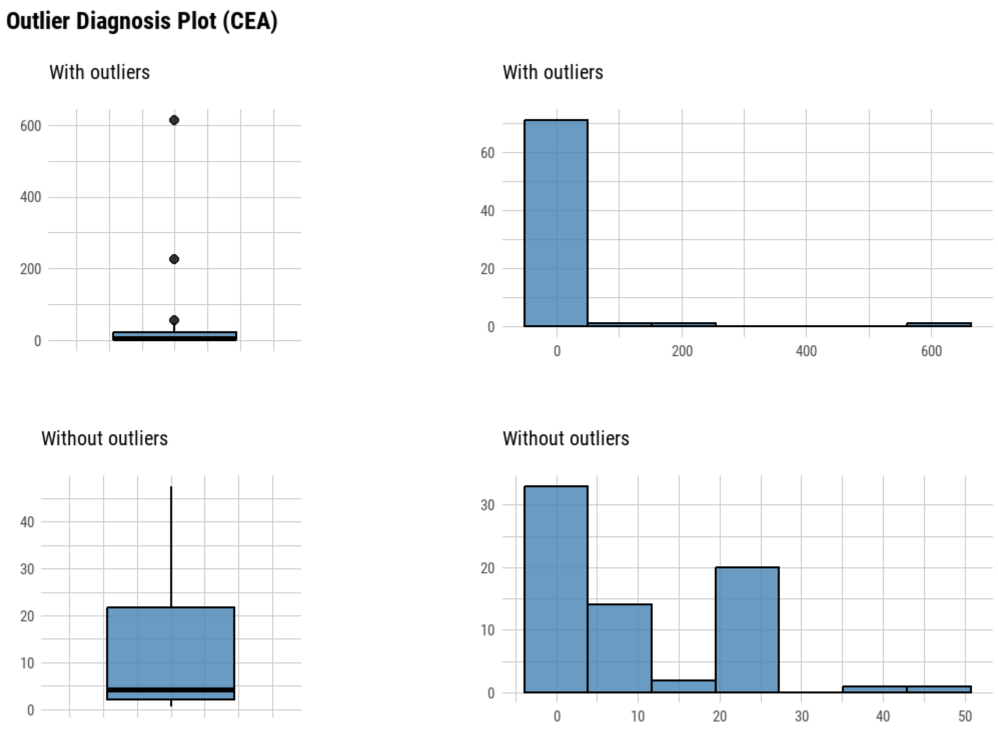

variable: SUVmax_SM

Measures	Values
Outliers count	3
Outliers ratio (%)	4.05%
Mean of outliers	1.81
Mean with outliers	1.043378
Mean without outliers	1.010986

Table 10: SUVmax_SM

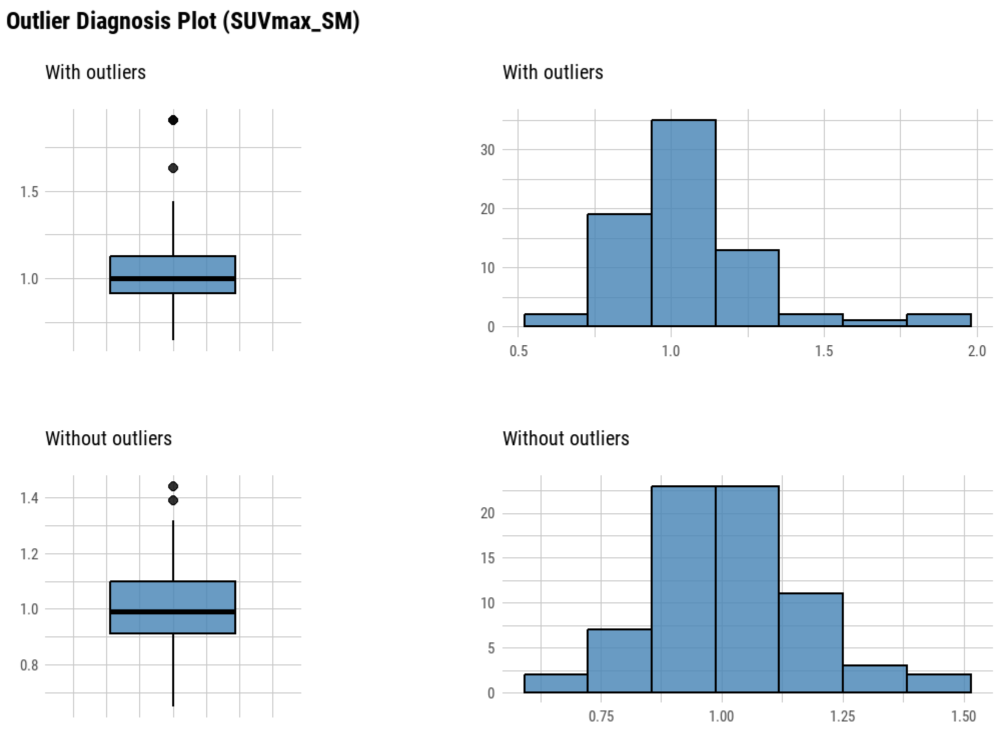

variable: SFA

Measures	Values
Outliers count	3
Outliers ratio (%)	4.05%
Mean of outliers	301.7667
Mean with outliers	138.9449
Mean without outliers	132.0651

Table 10: SFA

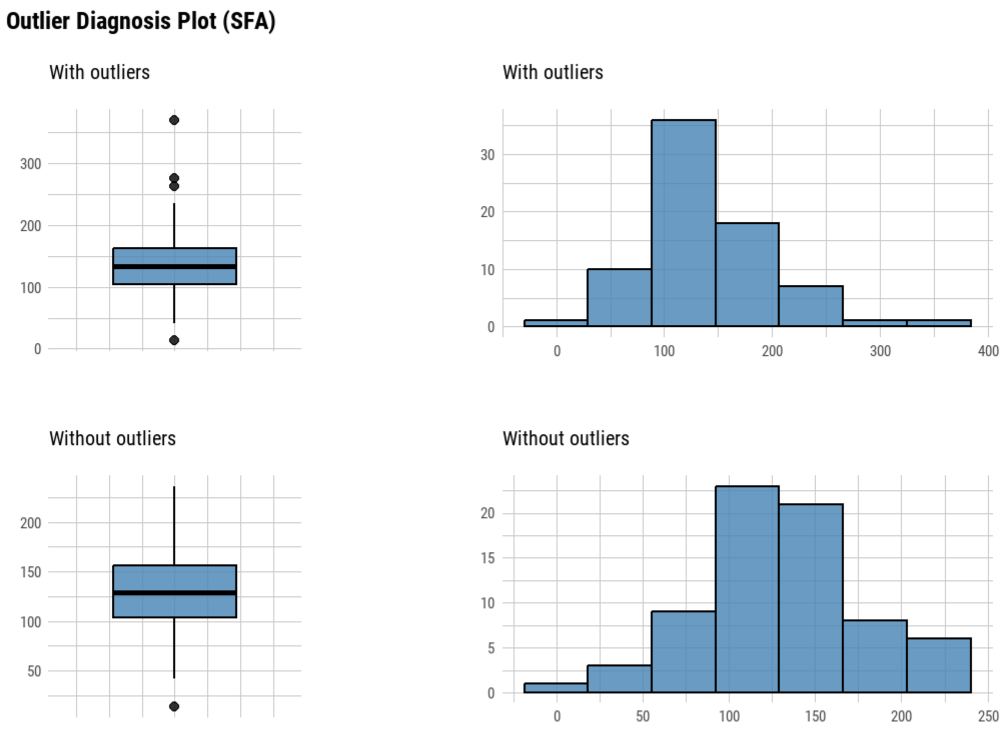

variable: time

Measures	Values
Outliers count	3
Outliers ratio (%)	4.05%
Mean of outliers	82
Mean with outliers	22.7973
Mean without outliers	20.29577

Table 10: time

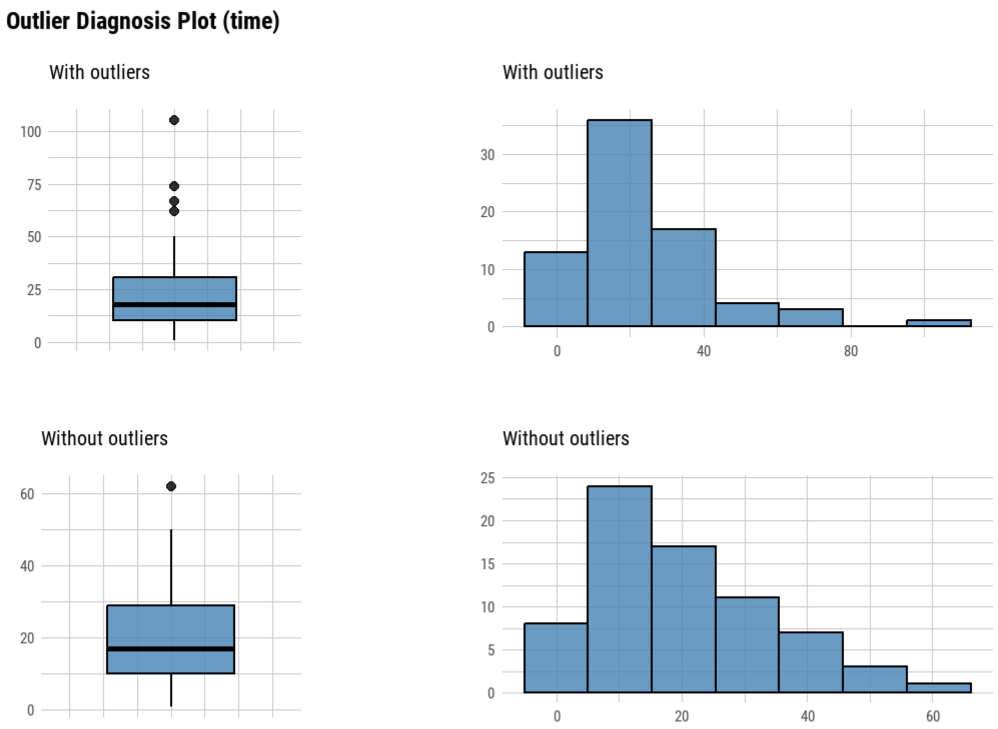

variable: BMI

Measures	Values
Outliers count	2
Outliers ratio (%)	2.7%
Mean of outliers	33.6
Mean with outliers	22.47338
Mean without outliers	22.16431

Table 10: BMI

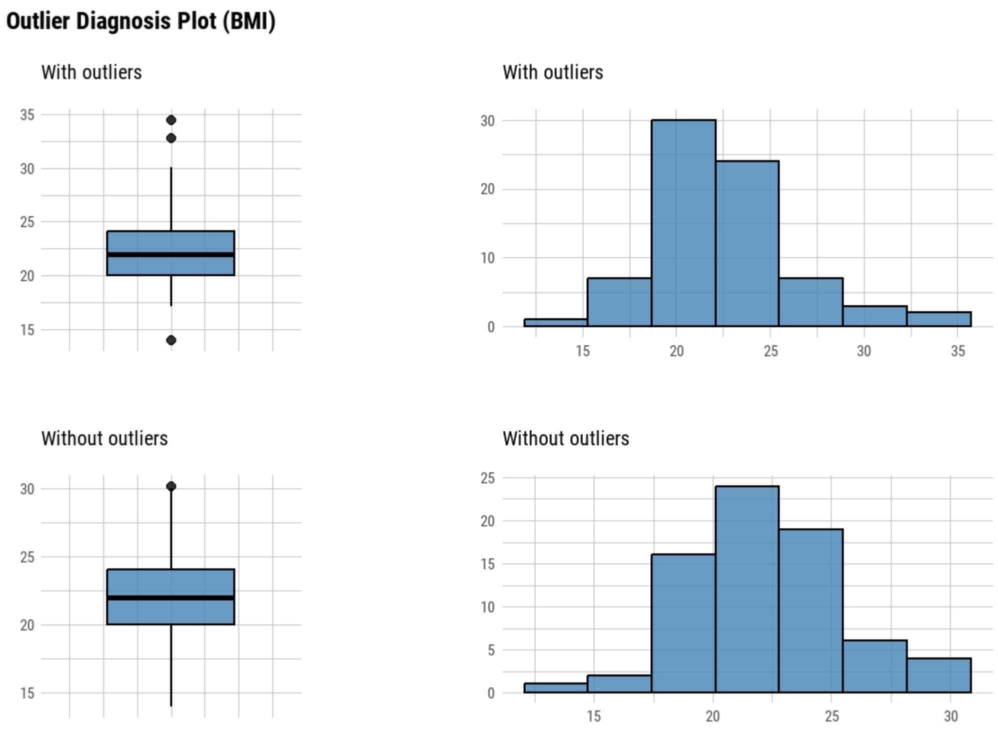

variable: Family.history

Measures	Values
Outliers count	2
Outliers ratio (%)	2.7%
Mean of outliers	1
Mean with outliers	1.972973
Mean without outliers	2

Table 10: Family.history

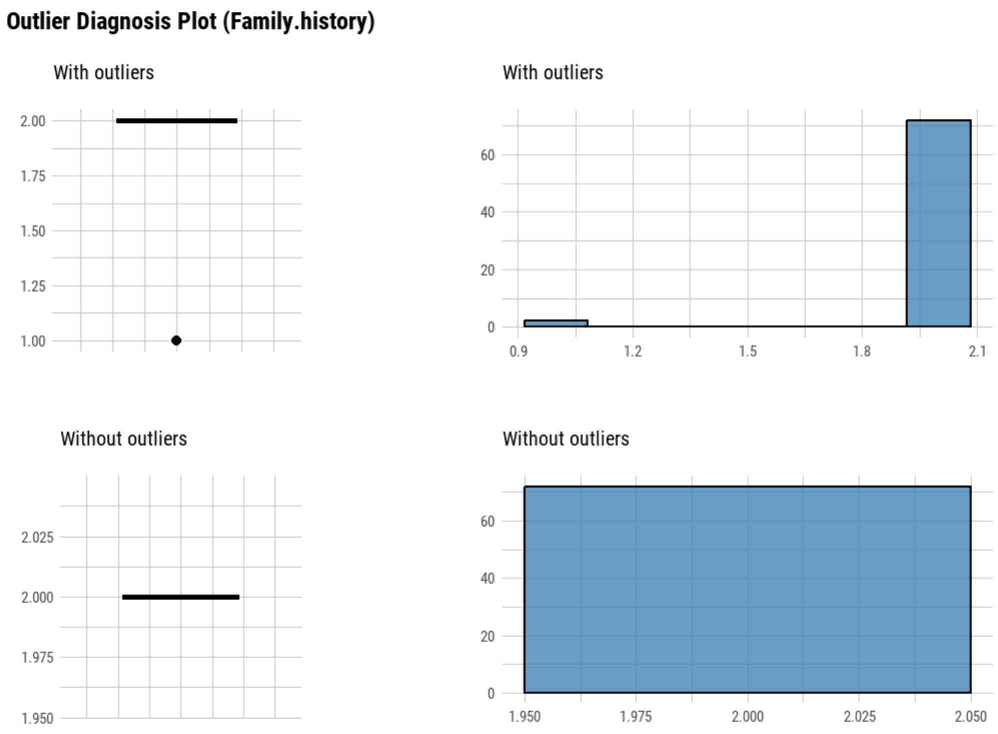

variable: SATI

Measures	Values
Outliers count	2
Outliers ratio (%)	2.7%
Mean of outliers	126.2041
Mean with outliers	56.47083
Mean without outliers	54.5338

Table 10: SATI

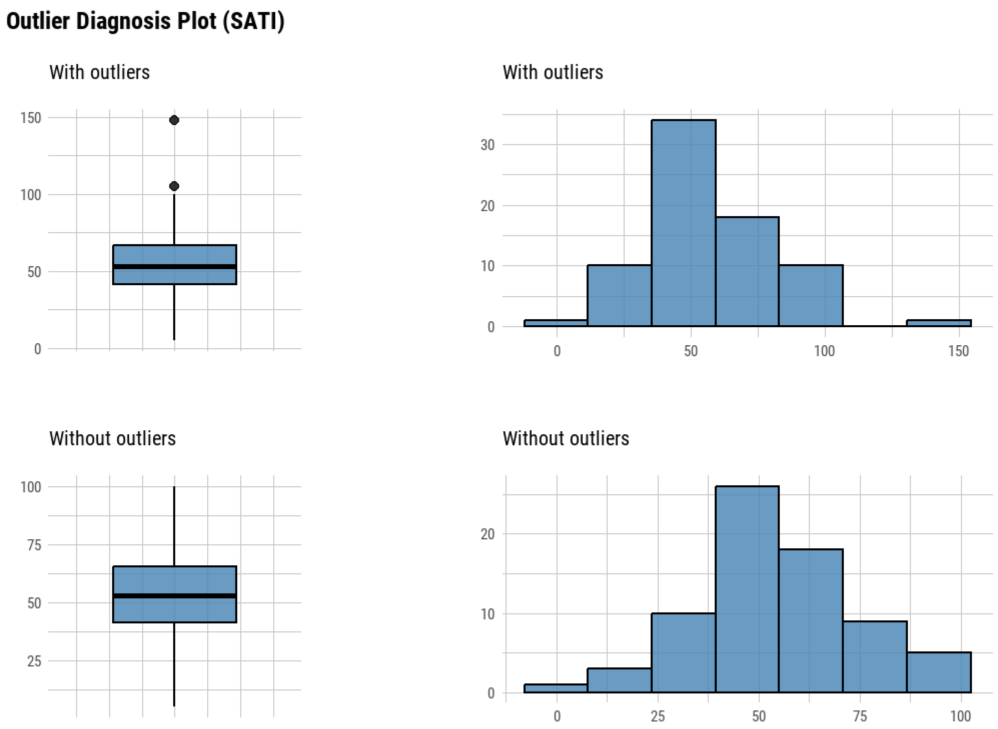

variable: SMI

Measures	Values
Outliers count	2
Outliers ratio (%)	2.7%
Mean of outliers	58.93779
Mean with outliers	39.69553
Mean without outliers	39.16102

Table 10: SMI

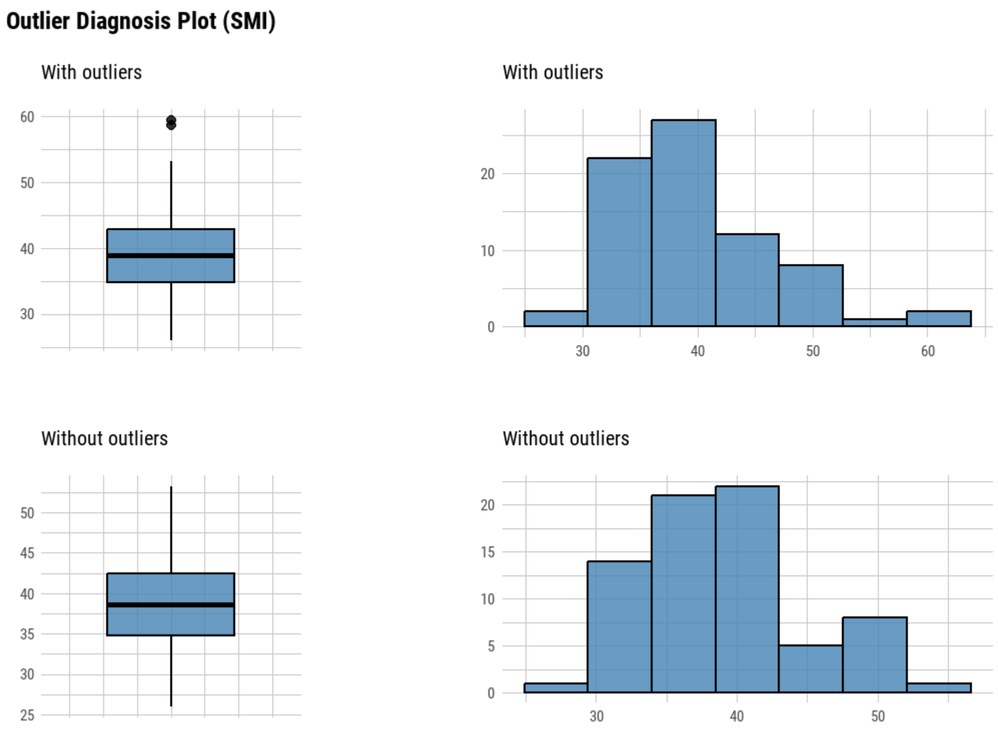

variable: SUVmean_VAT

Measures	Values
Outliers count	1
Outliers ratio (%)	1.35%
Mean of outliers	1.1
Mean with outliers	0.5617568
Mean without outliers	0.5543836

Table 10: SUVmean_VAT

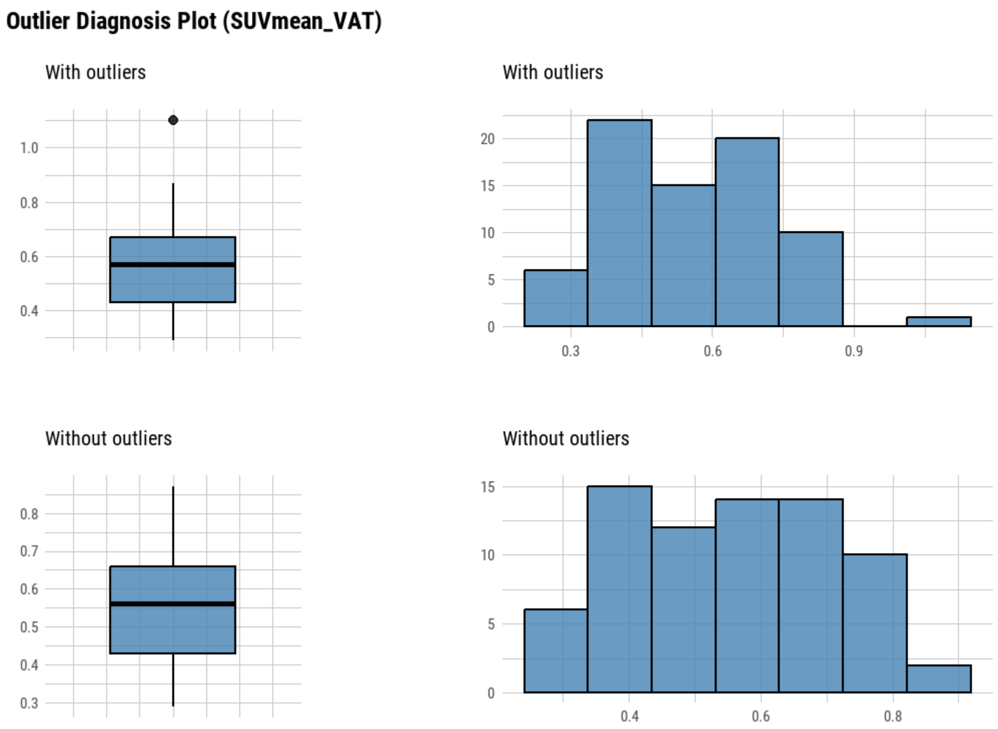

variable: SUVmax_VAT

Measures	Values
Outliers count	1
Outliers ratio (%)	1.35%
Mean of outliers	1.95
Mean with outliers	0.9439189
Mean without outliers	0.930137

Table 10: SUVmax_VAT

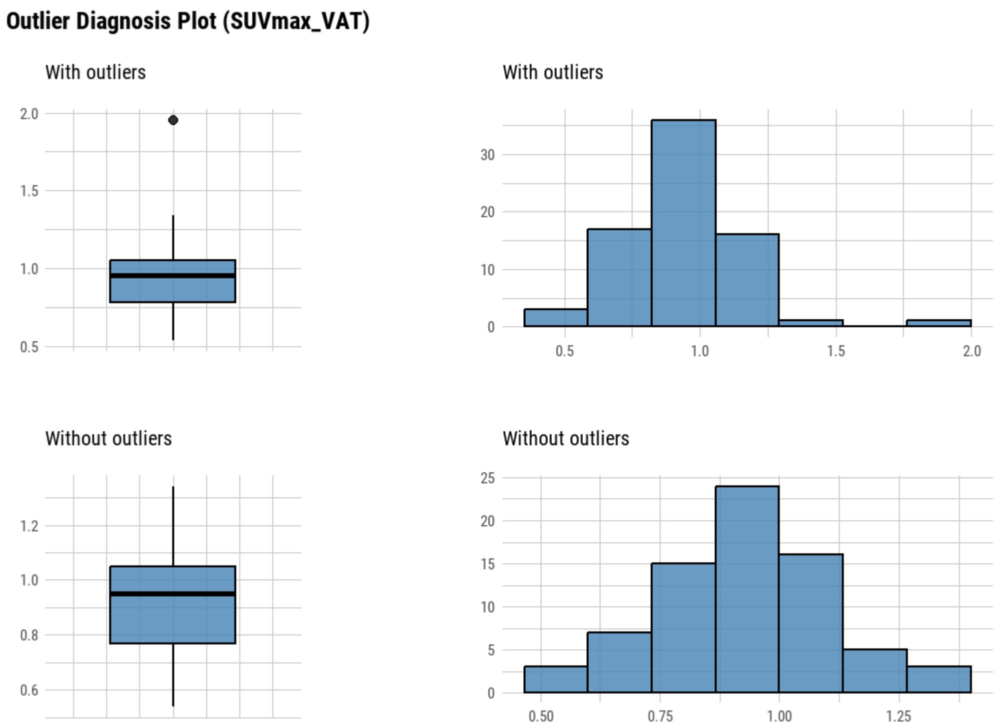

variable: SUVmax_SAT

Measures	Values
Outliers count	1
Outliers ratio (%)	1.35%
Mean of outliers	1.1
Mean with outliers	0.5072973
Mean without outliers	0.4991781

Table 10: SUVmax_SAT

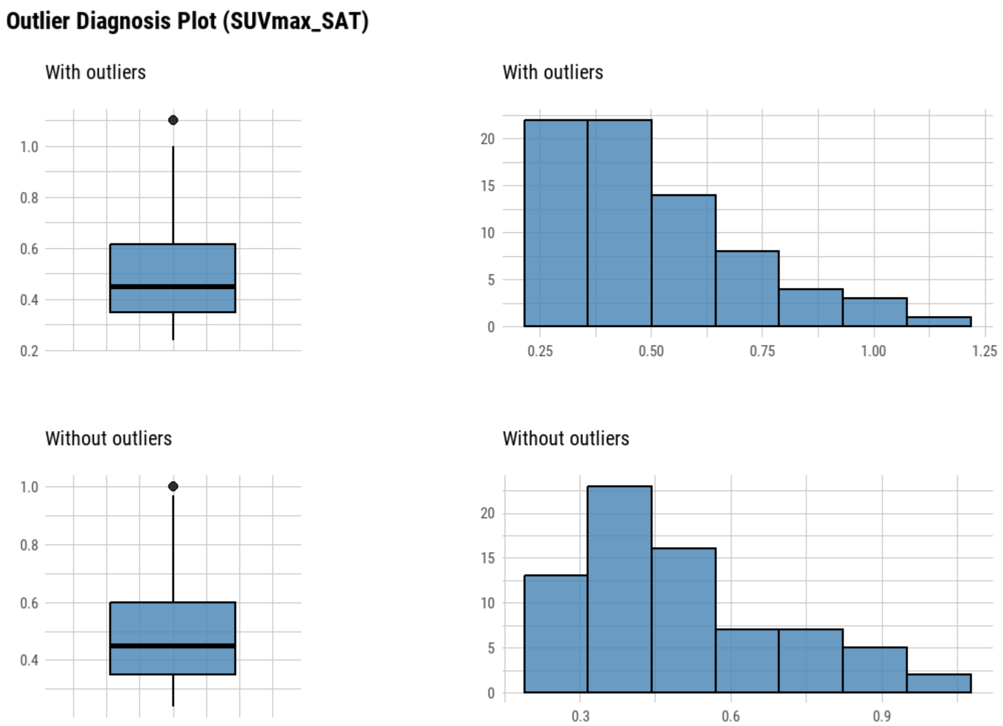

variable: SUVmean_SM

Measures	Values
Outliers count	1
Outliers ratio (%)	1.35%
Mean of outliers	1.13
Mean with outliers	0.637027
Mean without outliers	0.630274

Table 10: SUVmean_SM

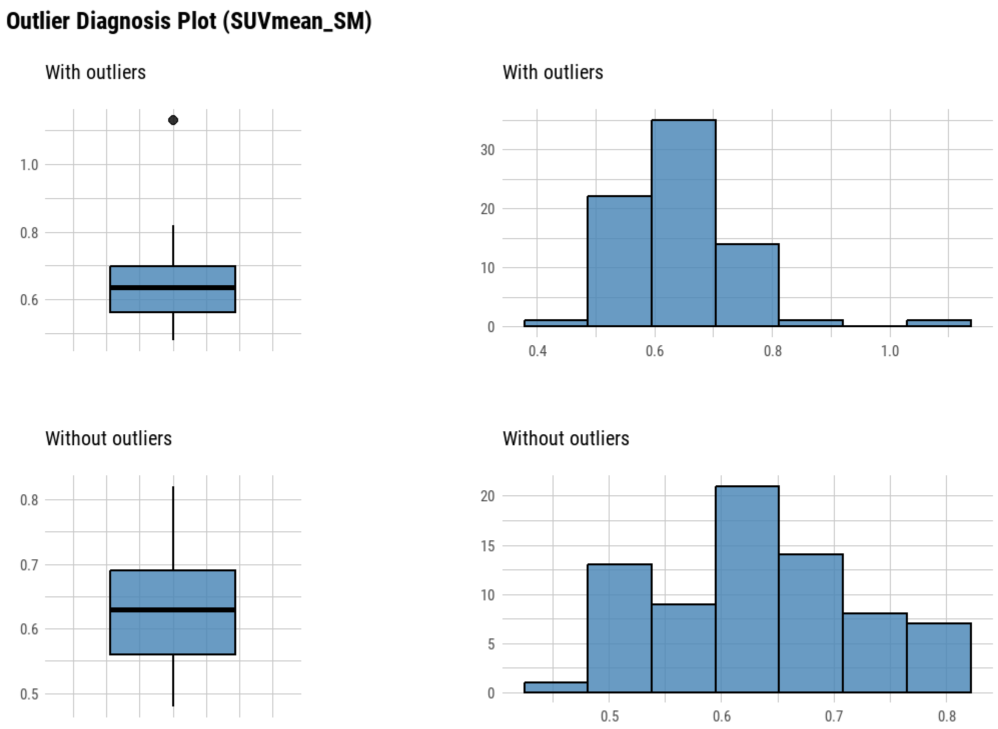

variable: SUVmax_VAT_SAT

Measures	Values
Outliers count	1
Outliers ratio (%)	1.35%
Mean of outliers	6.290323
Mean with outliers	2.13831
Mean without outliers	2.081433

Table 10: SUVmax_VAT_SAT

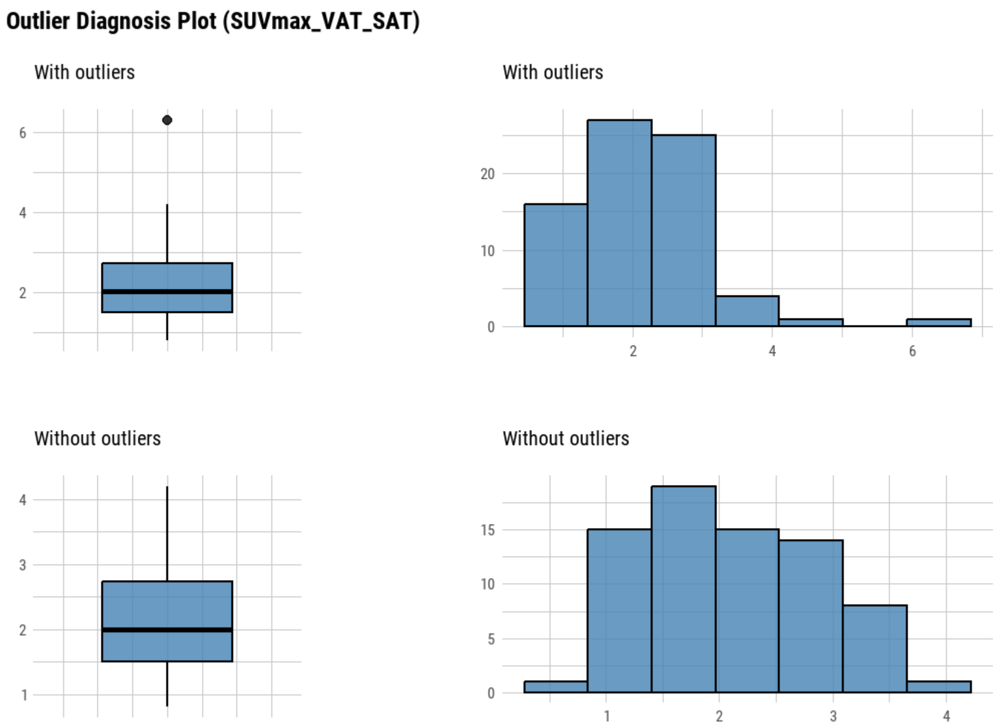

variable: VFA

Measures	Values
Outliers count	1
Outliers ratio (%)	1.35%
Mean of outliers	255.8
Mean with outliers	84.4435
Mean without outliers	82.09615

Table 10: VFA

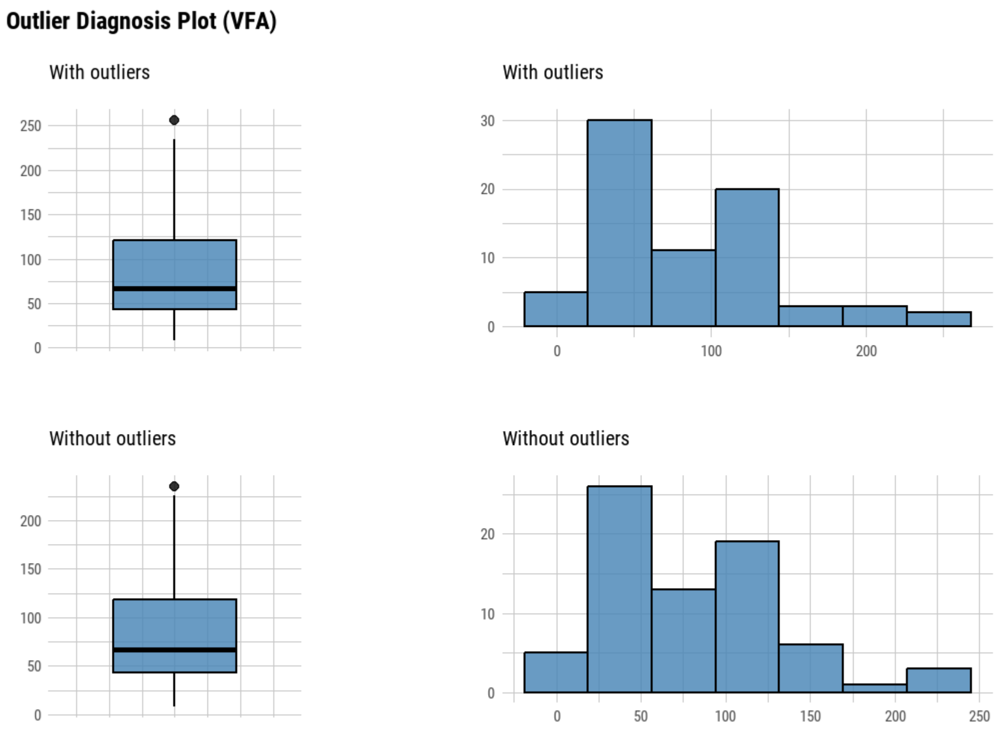

variable: VATI

Measures	Values
Outliers count	1
Outliers ratio (%)	1.35%
Mean of outliers	99.92188
Mean with outliers	34.20951
Mean without outliers	33.30934

Table 10: VATI

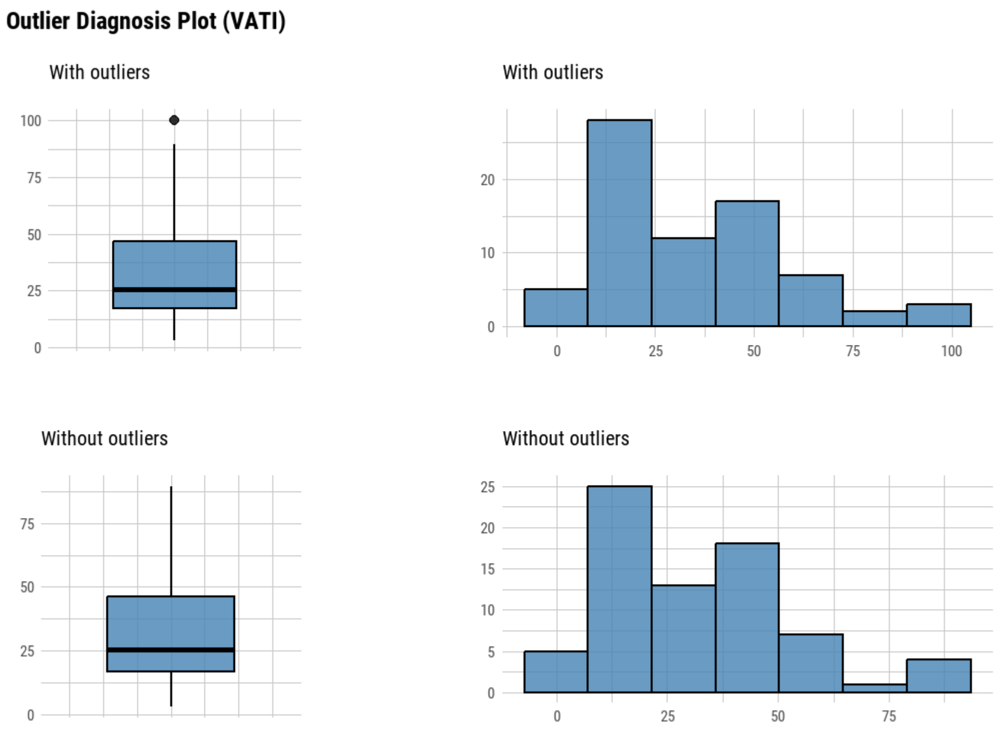

Supplement: Supplementary file 1 [file DataSheet1.pdf]
