# Supplementary material for: Factors associated with clinical outcomes of breast cancer based on glucose metabolic activity of subcutaneous adipose tissue
Source: Front Oncol. 2026 Feb 23;16:1722085. doi: 10.3389/fonc.2026.1722085 (PMC12967984; doi:10.3389/fonc.2026.1722085)
Supplement: Supplementary file 2 [file DataSheet2.docx]

# **
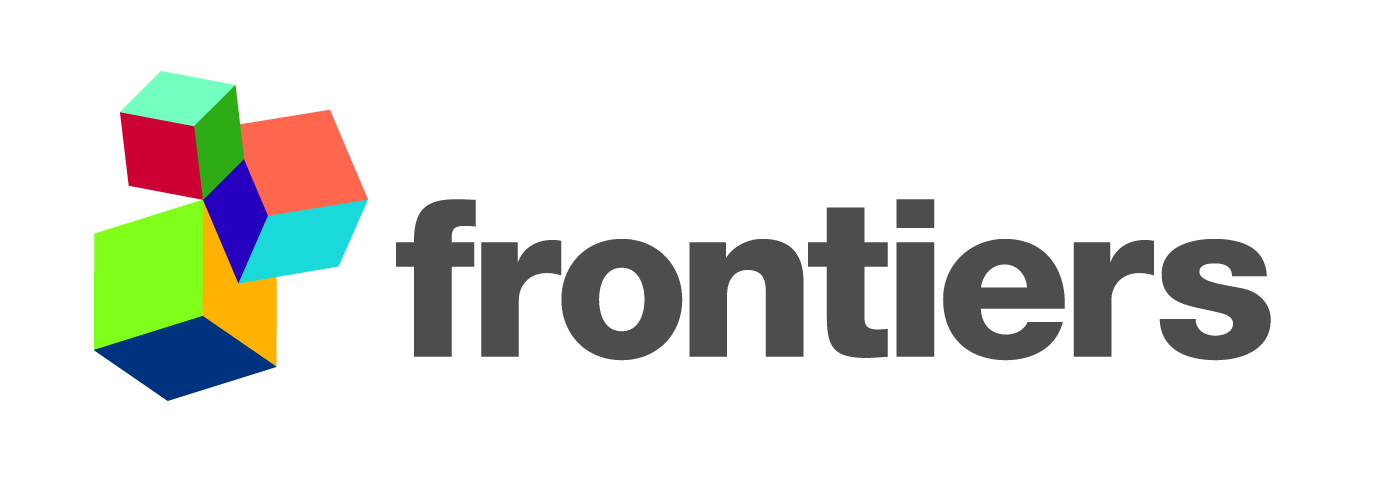
**

# Factors associated with clinical outcomes of breast cancer based on glucose metabolic activity of subcutaneous adipose tissue


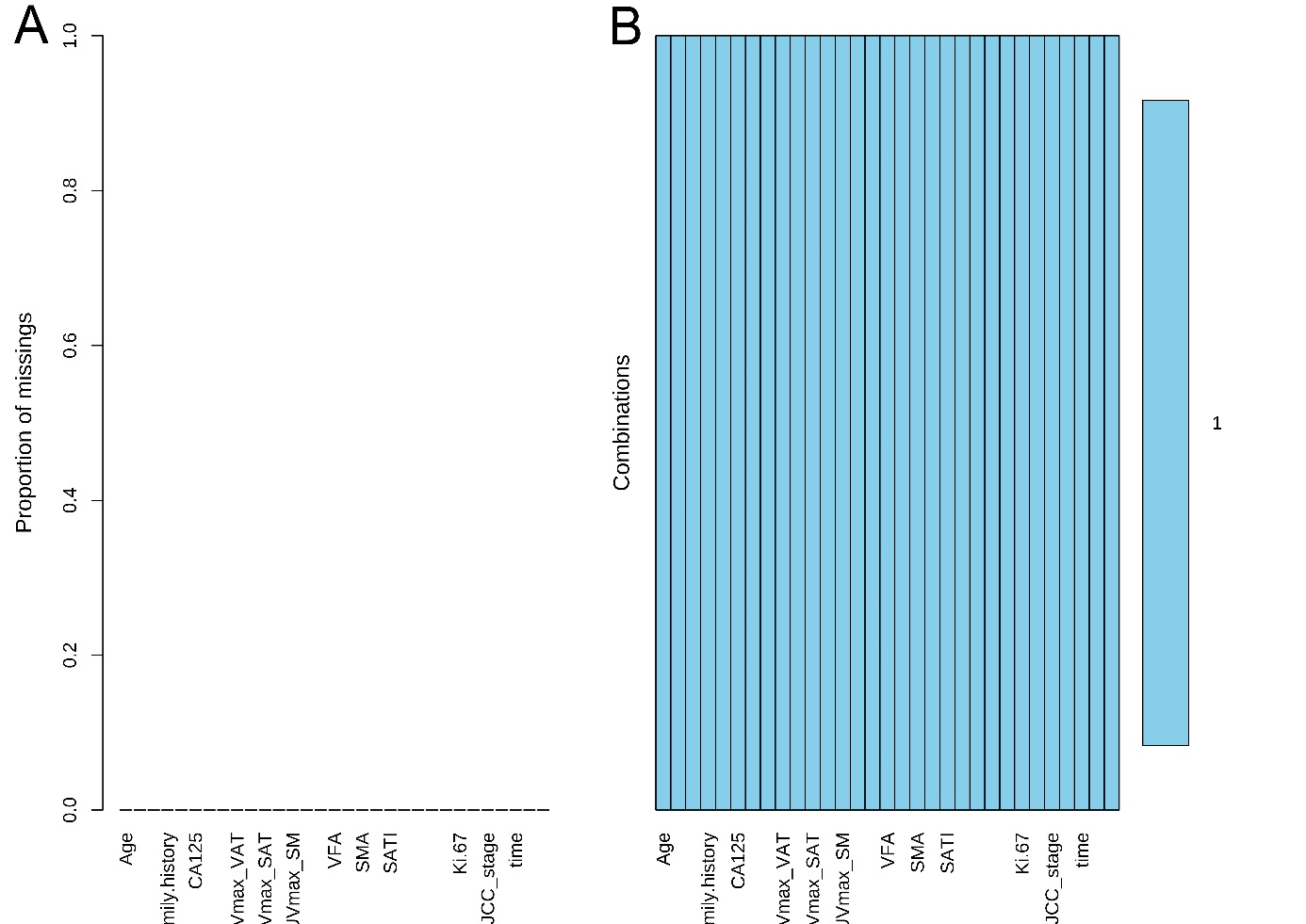


**Figure S1.** Aggregations plots for the missing values in the breast cancer dataset.

Missing values overall displayed in the Figure S1.
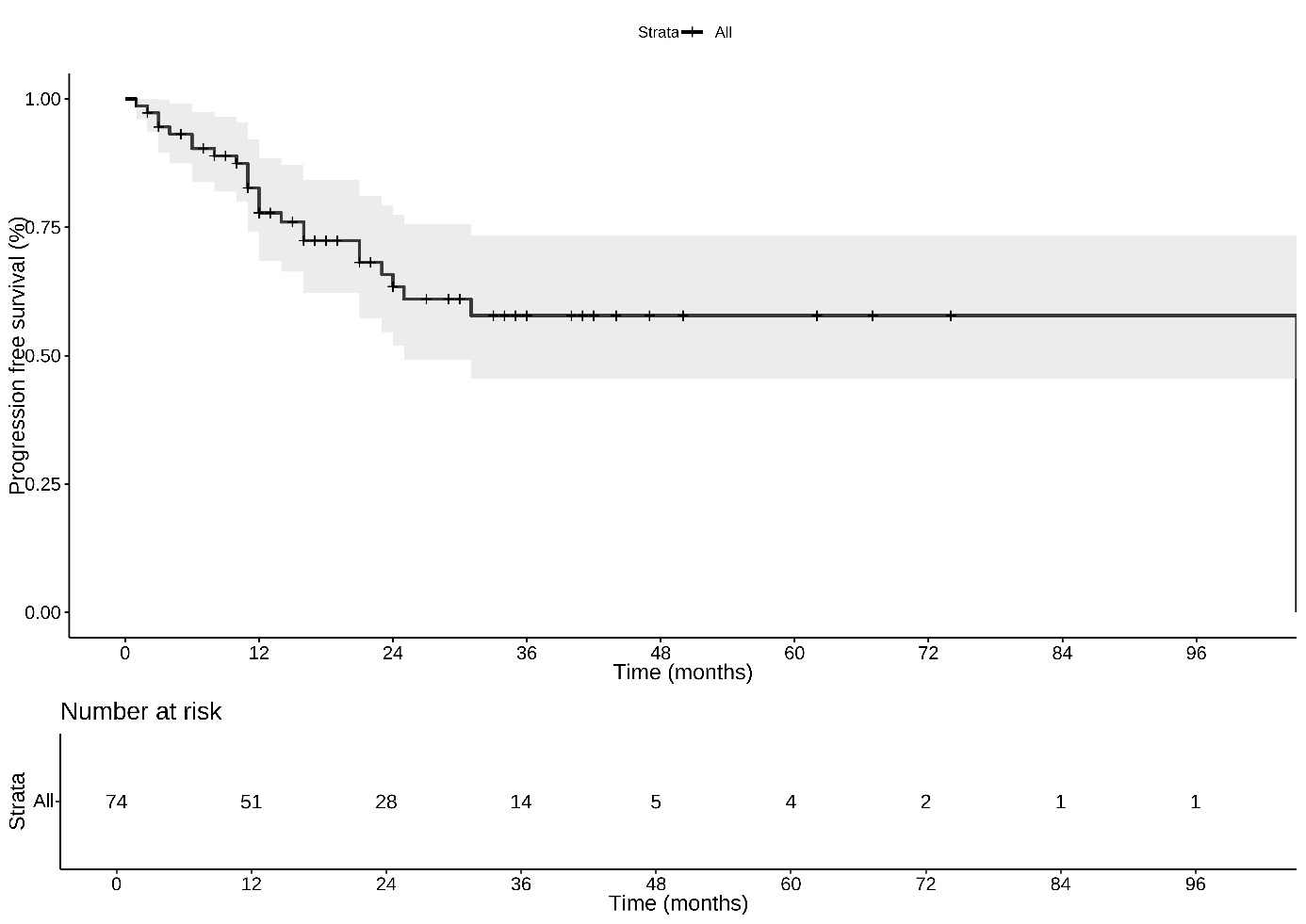


**Figure S2.** Drawing the overview survival curve for the breast cancer dataset.

An overview of the progression-free survival curve in the dataset (Figure S2).


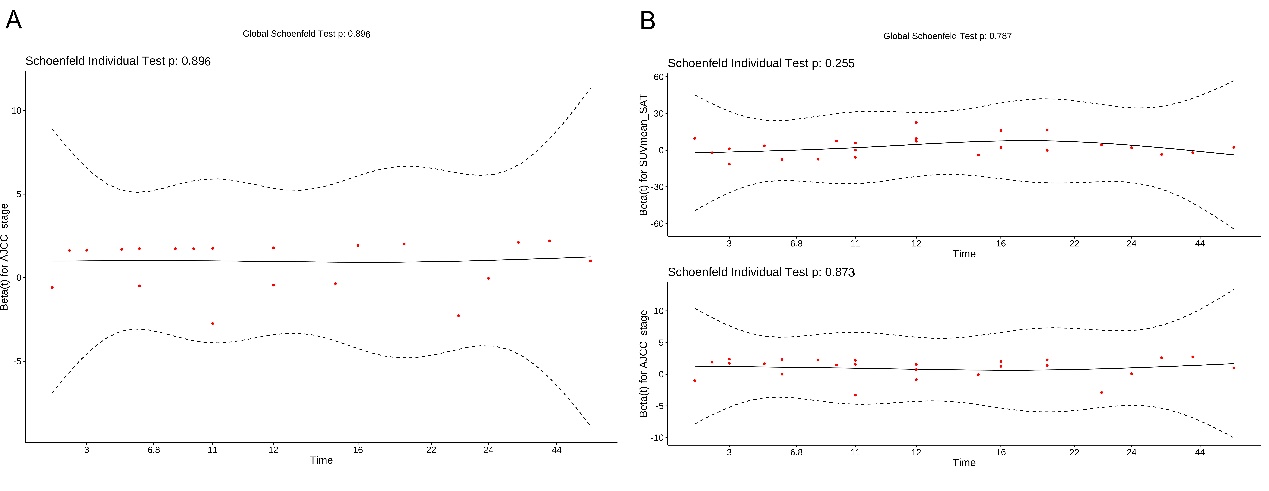


**Figure S3.** Testing the proportional hazards assumption of the two Cox regression models with the candidate variables using the Schoenfeld residuals method.

In the figureS3 graphs of the scaled Schoenfeld residuals against the transformed time, the solid line is a smoothing spline fit to the plot, with the dashed lines representing a ± 2-standard-error band around the Cox regression.


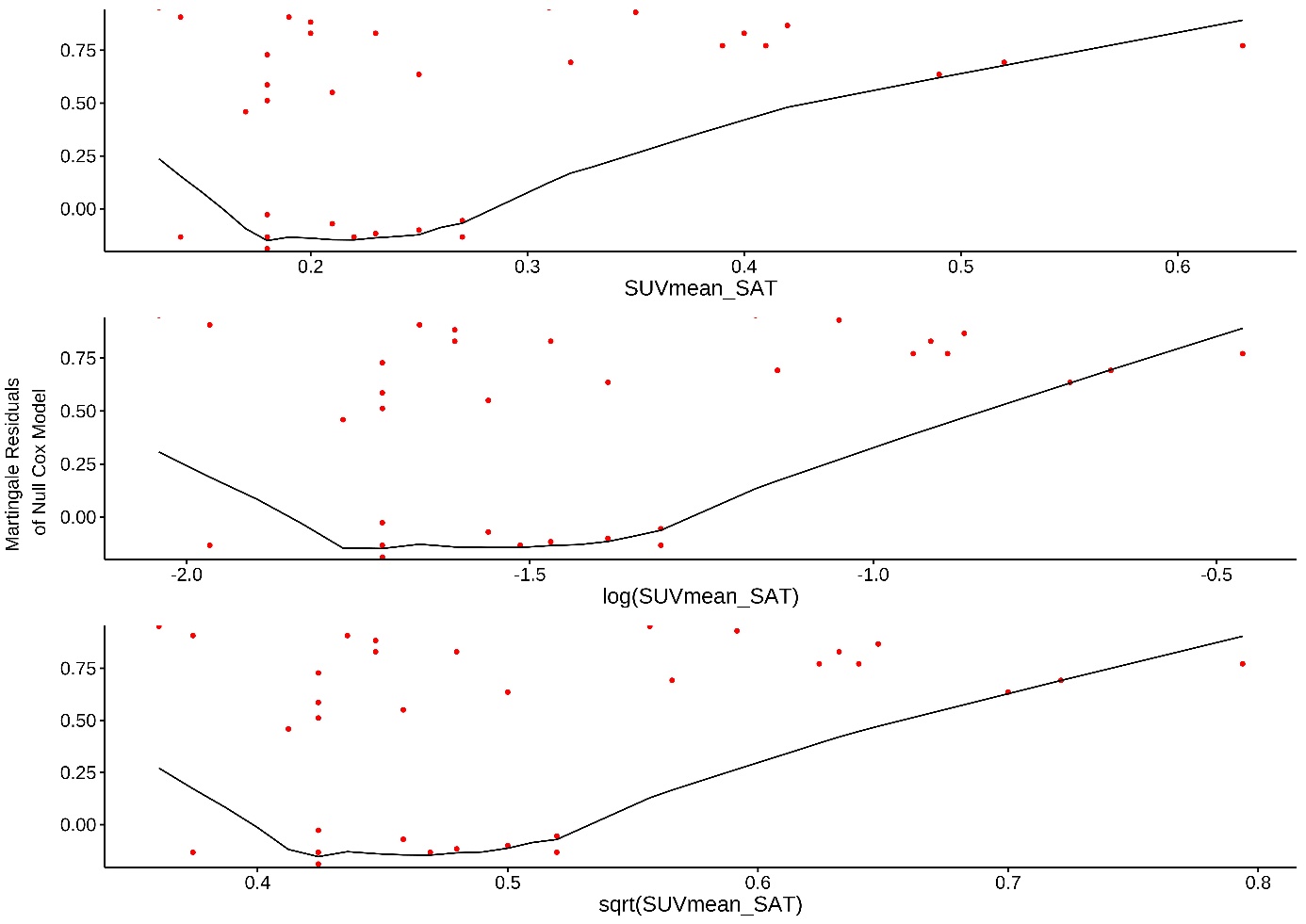


**Figure S4.** Displaying the continuous variable SUVmean_SAT against martingale residuals of null Cox proportional hazards model.

We plotted the Martingale residuals against continuous covariates SUVmean_SAT to detect nonlinearity or, in other words, to assess the functional form of a covariate. For a given continuous covariate, patterns in the plot may suggest that the variable SUVmean_SAT is not properly fit in Figure S4.


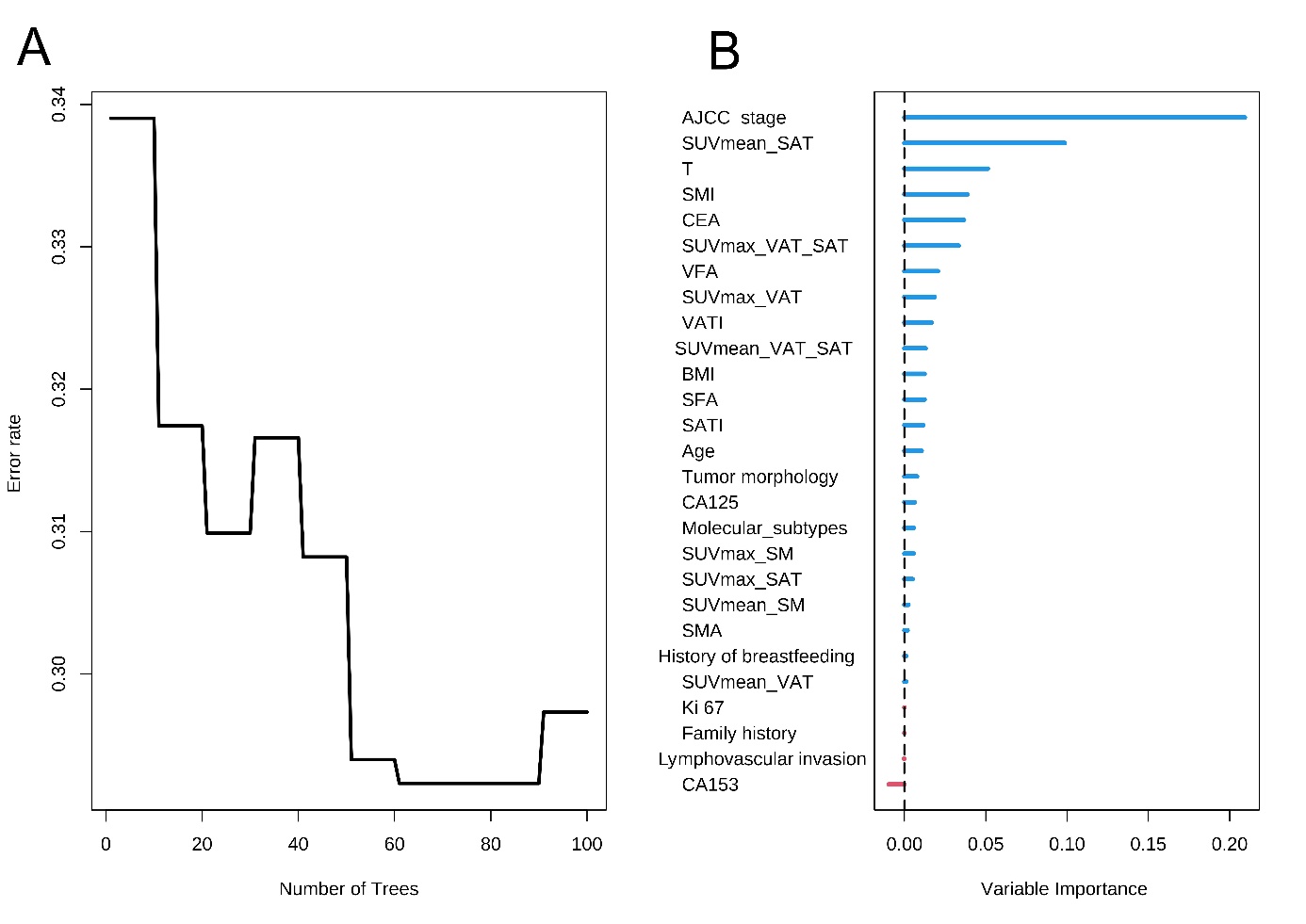


**Figure S5.** Fast unified random forests for survival analysis and the computing variable importance ranking.

In Figure S5, the variable importance in the survival random forest method was calculated using out-of-bag data in the way that assigns cases to the anti (opposite) split, when that variable is truly removed from the tree growing process.

**Table S1.** Significant cutoff value of SUVmean_SAT continuous variable for Cox regression.

| **cutoff points** | **n** | **y** | **HR** | ***p* value** |
| --- | --- | --- | --- | --- |
| 0.27 | 57/17 | 13/12 | 3.643 | 0.002 |
| 0.31 | 59/15 | 14/11 | 3.450 | 0.003 |
| 0.25 | 52/22 | 12/13 | 3.131 | 0.060 |
| 0.26 | 54/20 | 13/12 | 3.048 | 0.007 |
| 0.24 | 47/27 | 11/14 | 2.532 | 0.024 |
| 0.23 | 46/28 | 14/11 | 3.450 | 0.035 |
| *n* = patient numbers, *y* = progession patient numbers, *HR* = Hazard Ratio | | | | |

**Table S2.** Hazard ratios (with 95% CIs) of candidate variables and progression-free survival among patients with breast cancer.

|  | ***β*-Coefficient** | **HR** | **95% CIs** | ***p* value** |
| --- | --- | --- | --- | --- |
| **Model 1** |  |  |  |  |
| AJCC stage | 1.215 | 3.369 | 1.826, 6.216 | < 0.001 |
| **Model 2** |  |  |  |  |
| AJCC stage | 1.085 | 2.960 | 1.605, 5.458 | < 0.001 |
| SUVmean_SAT | 0.824 | 2.279 | 0.971, 5.350 | 0.045 |
| *HR* = Hazard Ratio, *CIs* = Confidence Intervals | | | | |
